# Usage of the Brief Job Stress Questionnaire: A Systematic Review of a Comprehensive Job Stress Questionnaire in Japan from 2003 to 2021

**DOI:** 10.3390/ijerph20031814

**Published:** 2023-01-18

**Authors:** Kazuhiro Watanabe, Kotaro Imamura, Hisashi Eguchi, Yui Hidaka, Yu Komase, Asuka Sakuraya, Akiomi Inoue, Yuka Kobayashi, Natsu Sasaki, Kanami Tsuno, Emiko Ando, Hideaki Arima, Hiroki Asaoka, Ayako Hino, Mako Iida, Mai Iwanaga, Reiko Inoue, Yasumasa Otsuka, Akihito Shimazu, Norito Kawakami, Akizumi Tsutsumi

**Affiliations:** 1 Department of Public Health, Kitasato University School of Medicine, 1-15-1 Kitazato, Minami-ku, Sagamihara 252-0374, Japan; 2 Department of Digital Mental Health, Graduate School of Medicine, The University of Tokyo, 7-3-1 Hongo, Bunkyo-ku, Tokyo 113-0033, Japan; 3 Department of Mental Health, Institute of Industrial Ecological Sciences, University of Occupational and Environmental Health, 1-1 Iseigaoka, Yahatanishi-ku, Kitakyushu 807-8555, Japan; 4 Department of Mental Health, Graduate School of Medicine, The University of Tokyo, 7-3-1 Hongo, Bunkyo-ku, Tokyo 113-0033, Japan; 5 Institutional Research Center, University of Occupational and Environmental Health, 1-1 Iseigaoka, Yahatanishi-ku, Kitakyushu 807-8555, Japan; 6 Faculty of Social Policy & Administration, Hosei University, 4342 Aiharamachi, Machida, Tokyo 194-0298, Japan; 7 School of Health Innovation, Kanagawa University of Human Services, 3-25-10 Tonomachi, Kawasaki-ku, Kawasaki 210-0821, Japan; 8 Institute for Cancer Control, National Cancer Center, 5-1-1 Tsukiji, Chuo-ku, Tokyo 104-0045, Japan; 9 Department of Psychiatric Nursing, Graduate School of Medicine, The University of Tokyo, 7-3-1 Hongo, Bunkyo-ku, Tokyo 113-0033, Japan; 10 Department of Community Mental Health & Law, National Center of Neurology and Psychiatry, National Institute of Mental Health, 4-1-1 Ogawahigashi, Kodaira, Tokyo 187-0031, Japan; 11 Faculty of Human Sciences, University of Tsukuba, 3-29-1 Otsuka, Bunkyo-ku, Tokyo 112-0012, Japan; 12 Faculty of Policy Management, Keio University, 5322 Endo, Fujisawa 252-0882, Japan

**Keywords:** questionnaire, psychometric, workplace, job demands, job control, social support

## Abstract

The Brief Job Stress Questionnaire (BJSQ) is used widely in occupational health studies and practice. Summarizing scientific production based on measurement is crucial. This study aimed to systematically review observational studies that used the BJSQ and the New BJSQ to show their usability. A systematic search was conducted for studies investigating relationships between the BJSQ or the New BJSQ subscales and other validated measurements on 13 September 2021, in various literature databases. The BJSQ subscales, scoring methods, and other validated measurements in the studies were qualitatively summarized. In total, 145 published reports between 2003 and 2021 were included. Among the BJSQ subscales, job stressors (n = 95) such as quantitative job overload (n = 65) and job control (n = 64) were most often used. The subscales were utilized to investigate the relationships with several other measurements. Five reports used subscales from the New BJSQ. In the last two decades, the BJSQ and the New BJSQ help measure psychosocial factors (PF) at work and contribute to the publication of scientific papers in the occupational health field. This study would encourage the utilization of the questionnaires for future research and practice.

## 1. Introduction

### 1.1. Background and Previous Work

Conducting multidimensional identification, assessment, and control of psychosocial factors (PF) at work is important in psychosocial risk management for occupational safety and health. Exposure to psychosocial stressors at work leads to physical and mental health problems among workers. Scientific evidence has indicated that high job demands, low job control, low social support, effort–reward imbalance, and high job insecurity elevate the risk of coronary heart disease and mental disorders [1,2,3]. In practice, several countries and regions have guidance or standards, such as the Psychosocial Risk Management—European Framework (PRIMA-EF) [4], the United Kingdom (UK) Health and Safety Executive (HSE) management standards [5], the National Standard of Canada for Psychological Health and Safety [6], and standards by the International Organization for Standardization (ISO) [7], that emphasize the importance of assessing multidimensional risks related to PF at work.

Numerous questionnaires and scales are available to measure and identify multiple PF at work and used in both research and practice. For example, in UK HSE management standards [5], the indicator tool helps assess employee perceptions of six key stressor areas: demands, control, support, relationships, role, and organizational change. The Copenhagen Psychosocial Questionnaire (COPSOQ) [8,9,10] measures a broad range of PF, including stressors, health and well-being, and personality. The third version of COPSOQ covers eight domains and 26 dimensions using validated items [10]. The Generic Job Stress Questionnaire (GJSQ) from the United States of America National Institute for Occupational Safety and Health (USA NIOSH) also covers various job stressors, mental health, and personality [11]. The Korean Occupational Stress Scale (KOSS) was developed in Korea; it consists of eight subscales of job stressors: physical environment, job demand, insufficient job control, interpersonal conflict, job insecurity, organizational system, lack of reward, and occupational climate [12]. Multidimensional scales to measure PF at work in specific industries were also developed and reported such as for construction workers [13], teachers [14], nurses [15], and dentists [16].

### 1.2. Background in Japan

In Japan, the 57 items of the Brief Job Stress Questionnaire (BJSQ) was developed in 2000 [17] based on the GJSQ from the USA NIOSH [11], covering job stressors, stress responses, buffering factors (i.e., social support), and job satisfaction. Among job stressors, the BJSQ includes quantitative job overload (three items), qualitative job overload (three items), physical demands (one item), job control (three items), skill utilization (one item), interpersonal conflict (three items), poor physical environment (one item), suitable jobs (one item), and meaningfulness of work (one item). Stress responses include vigor (three items), anger-irritability (three items), fatigue (three items), anxiety (three items), depression (six items), and physical complaints (11 items). Buffering factors include support from supervisors (three items), coworkers (three items), and family and friends (three items). Job and life satisfaction are also measured by a single item for each. All items are rated on a four-point scale. The New BJSQ was developed in 2014, covering effort–reward imbalance, bullying, organizational factors, work–self balance, and positive outcomes [14]. Most subscales in the BJSQ and the New BJSQ showed acceptable levels of internal consistency, test–retest reliability, and structural validity [18].

In the last two decades, the BJSQ has been used widely for occupational health studies and practice and tests the associations with a broad range of outcomes, including biological markers. The New BJSQ has also been used in later studies. Recently, the Japanese government launched a new occupational health policy called the National Stress Check Program (NSCP); this policy mandates that workplaces with 50 or more employees conduct assessments of psychosocial stress in employees at least once a year [19]. This policy recommends the use of the BJSQ as a structured questionnaire for the assessment. Thereafter, the BJSQ has been used more frequently, and the publication of data measured by the BJSQ has increased rapidly.

### 1.3. Research Gaps and Objectives

However, no systematic review has reported on the usage of the BJSQ and the New BJSQ and the findings of studies that used these questionnaires. For the COPSOQ, systematic reviews for the usage of the measurements have already been reported [20,21], and the international scientific production was summarized. Moreover, a systematic review of the BJSQ and the New BJSQ is important to make a milestone of scientific production from these measurements. Additionally, the summary of published data measured using the BJSQ and the New BJSQ, including samples, subscales, and scoring methods, would be useful statistics for research and practice in occupational health in Japan. The correlates of the BJSQ and the New BJSQ would be useful for validating the questionnaires and accumulating scientific evidence of the association between PF at work and health. This study aimed to systematically review observational studies that used the BJSQ and the New BJSQ to show their usability. Published literature until 2021 was systematically reviewed using various databases. The BJSQ subscales, scoring methods, and other validated measurements were qualitatively summarized. This study significantly contributes to creating a new summary of the questionnaires and encouraging the utilization of the questionnaires in future research and practice in occupational health.

## 2. Materials and Methods

### 2.1. Study Design

This study was a systematic review of observational studies. The reporting in this study was conducted following the updated guideline for reporting systematic reviews (the Preferred Reporting Items for Systematic Reviews and Meta-Analysis (PRISMA) 2020 statement) [22]. The study protocol was registered at the University Hospital Medical Information Network Clinical Trials Registry (UMIN-CTR, ID R000045091) in Japan.

### 2.2. Eligibility Criteria

For the systematic review, the authors included studies that (1) adopted observational study design; (2) sampled workers; (3) used at least one subscale of the BJSQ or the New BJSQ; (4) used other validated measurements and tested associations between the BJSQ or the New BJSQ subscales and other measurements; (5) were written in English or Japanese; and (6) were peer-reviewed. Studies were also included if they used the BJSQ and the New BJSQ subscales as non-primary variables and investigated the associations in preliminary analyses. Those studies that used other single-item measurements (e.g., smoking status), except for shift work, working hours, sleeping hours, subjective views of health, subjective well-being, and subjective satisfaction, were excluded.

### 2.3. Information Sources and Search Strategy

A systematic search of the literature on 13 September 2021 was conducted on databases such as MEDLINE (PubMed), EMBASE, PsycINFO/ARTICLES, and Japan Medical Abstract Society. For search terms, “brief job stress questionnaire” OR “BJSQ” was used, and no filter/limit was applied for any of the databases.

### 2.4. Study Selection and Data Collection Process

Identified records were managed in a Microsoft Excel (Washington, DC, USA) file. One investigator sorted the records by title and removed duplicates. Subsequently, each record was assigned to two reviewers from among 13 investigators. The investigators independently judged whether a record met the inclusion criteria of the systematic review. Records judged as not eligible by both of the two contributors were excluded, and other records were sought for retrieval of full texts. The full texts were judged by two independent reviewers, different from the initial screening, from 18 investigators. Reports assessed as eligible by both reviewers were included for review. When two investigators had inconsistent judgment at this full-text review stage, an agreement was reached through discussions with the project directors. When a report was excluded at this stage, the primary reasons for exclusion were recorded.

One of the reviewers of each study collected data from that study. The data were then reviewed by KW. The collected data included the names of the first authors, study design (cross-sectional or longitudinal), samples, subscales of the BJSQ and the New BJSQ, scoring methods of the BJSQ and the New BJSQ, and other validated measurements.

### 2.5. Data Synthesis and Analysis

Since this study aimed to summarize the usage of the BJSQ and the New BJSQ, no statistical data synthesis was conducted. Assessments of risk of bias within individual studies, heterogeneity, reporting bias, and certainty of evidence were not required to be conducted either. The collected data in the text and tabulation were qualitatively summarized. In addition, the number of subscales of the BJSQ and the New BJSQ and other measurements used were counted and visually placed, classifying them into five categories according to the job stress model [11]: (1) job stressors or exposures that relate to work conditions which lead to stress responses; (2) health-related outcomes including physiological and psychological responses; (3) work-related outcomes such as job satisfaction, job performance, and burnout; (4) individual and behavioral factors that modify the associations between job stressors and outcomes; and (5) buffering and non-work factors such as social support.

## 3. Results

### 3.1. Study Selection

**Figure 1:**
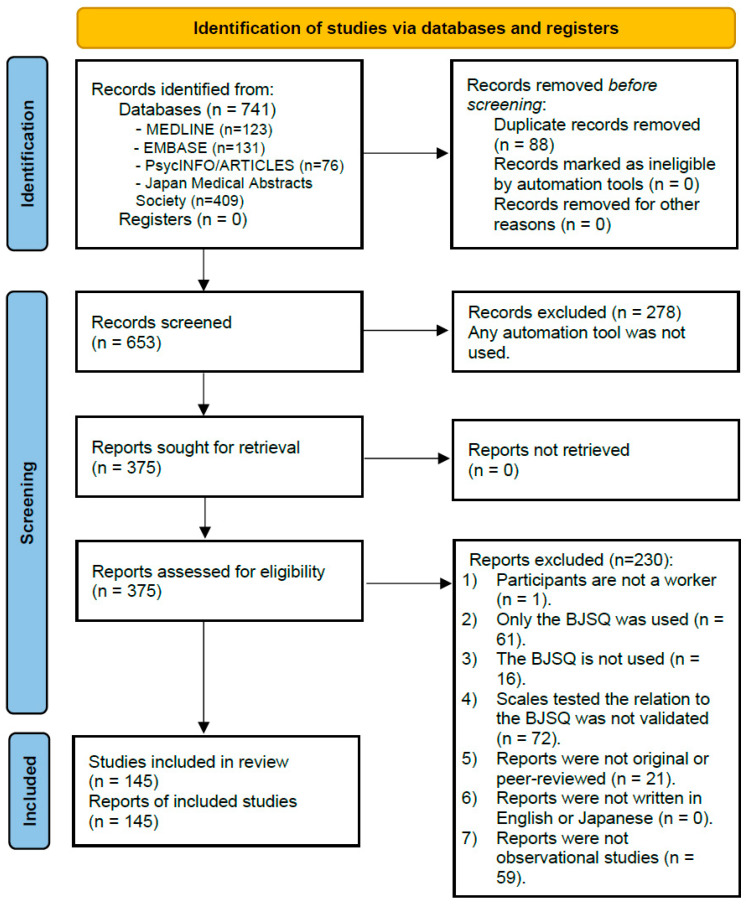
PRISMA 2020 flow diagram for new systematic reviews which included searches of databases and registers only.

Figure 1 illustrates the selection process of this systematic review. A systematic search of databases resulted in 741 hits. After the initial screening and the full-text review by the independent reviewers, 145 reports published from 2003 to 2021 were included in this systematic review [23,24,25,26,27,28,29,30,31,32,33,34,35,36,37,38,39,40,41,42,43,44,45,46,47,48,49,50,51,52,53,54,55,56,57,58,59,60,61,62,63,64,65,66,67,68,69,70,71,72,73,74,75,76,77,78,79,80,81,82,83,84,85,86,87,88,89,90,91,92,93,94,95,96,97,98,99,100,101,102,103,104,105,106,107,108,109,110,111,112,113,114,115,116,117,118,119,120,121,122,123,124,125,126,127,128,129,130,131,132,133,134,135,136,137,138,139,140,141,142,143,144,145,146,147,148,149,150,151,152,153,154,155,156,157,158,159,160,161,162,163,164,165,166,167]. Of the included reports, 102 had digital object identifiers, and the main text of 52 reports was written in Japanese.

A total of 230 reports were excluded at the full-text review stage, although some of them might have met the inclusion criteria. For example, Eguchi et al. [168] investigated the association between psychological stress response measured by the BJSQ and workplace occupational mental health (OMH) and related activities. However, the items of OHM activities were derived from a paper by the Japanese government and were not psychometrically validated. Kawada and Otsuka [169] conducted a longitudinal study to examine changes in job stress and job satisfaction using the BJSQ. However, they only reported the associations among the subscales of the BJSQ, not with other validated measurements. Iguchi [170] examined the associations among job demands, job resources, and turnover intention among public health nurses using the BJSQ and the New BJSQ. However, this study conducted a factor analysis for the subscales and conceptualized new variables in the analysis. Some studies used the BJSQ overseas: China, India, and the USA [171,172,173]. These studies did not report the validity of the translated version of the BJSQ.

### 3.2. Study Characteristics

A summary of the included studies is shown in the Table A1. Most studies were conducted cross-sectionally (n = 116) [52,53,54,55,56,57,58,59,60,61,62,63,64,65,66,67,68,69,70,71,72,73,74,75,76,77,78,79,80,81,82,83,84,85,86,87,88,89,90,91,92,93,94,95,96,97,98,99,100,101,102,103,104,105,106,107,108,109,110,111,112,113,114,115,116,117,118,119,120,121,122,123,124,125,126,127,128,129,130,131,132,133,134,135,136,137,138,139,140,141,142,143,144,145,146,147,148,149,150,151,152,153,154,155,156,157,158,159,160,161,162,163,164,165,166,167], while the remaining were longitudinal studies (n = 29) [23,24,25,26,27,28,29,30,31,32,33,34,35,36,37,38,39,40,41,42,43,44,45,46,47,48,49,50,51]. The sample size ranged from 18 [83] to 69,805 [60]. In the included studies, recruitment of the participants was conducted from private companies (n = 59), hospitals (n = 42), nursing or welfare facilities (n = 13), healthcare centers (n = 7), web surveys (n = 5), public sectors (n = 5), existing cohorts (n = 4), fire defense stations/headquarters (n = 4), and a convenience sample of faculty staff members or alumni of universities (n = 6).

### 3.3. Used Subscales and Other Measurements

**Figure 2:**
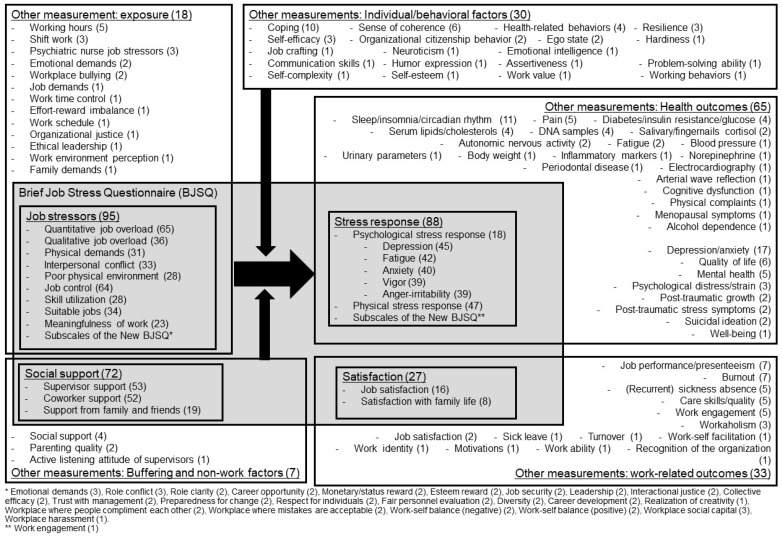
Used subscales from the BJSQ, the New BJSQ, and other measurements in the included studies. Note. Parenthesis in each subscale shows the number of times the measurements have been used.

Figure 2 shows the list of used subscales from the BJSQ, the New BJSQ, and other measurements in the included studies. Parenthesis in each subscale shows the number of times the measurements have been used.

For the subscales of the BJSQ, job stressors (n = 95), especially quantitative job overload (n = 65) and job control (n = 64), were most often used; stress responses (n = 88) and social support (n = 72) were frequently used as well. Most of the studies referred to the job stress model from the US NIOSH [11] or the job demands–control model by Karasek [174,175]. For example, Izawa et al. [95] used the subscales of quantitative job overload and job control from the BJSQ, calculated the job strain index by dividing quantitative job overload by job control, and investigated an association with cortisol levels in fingernails. Hidaka et al. [54] also adopted job strain through quantitative job overload and job control and social support from supervisors and coworkers of the BJSQ. They indicated those significant associations with health-related quality of life among Japanese workers. Stress responses were often used as the health outcomes explained by PF at work. A two-year follow-up study by Taniguchi et al. [37] investigated the association between workplace bullying and harassment and stress responses of the BJSQ among care workers at welfare facilities for the elderly. They reported multiple types of bullying and harassment were positively associated with psychological stress response at the follow-up. Shimazu and de Jonge [48] also used stress responses from the BJSQ as an outcome of the effort–reward imbalance and reported the reciprocal associations in a three-wave panel survey. Satisfaction (n = 27) was mainly utilized for examining associations with other health outcomes. Inoue et al. [28] investigated the prospective association between job satisfaction of the BJSQ and long-term sickness absence. They indicated that workers who perceived job dissatisfaction had a significantly higher risk of long-term sickness absence; however, after additionally adjusting for the psychosocial work environment, this association was weakened and was no longer significant.

The subscales of the BJSQ were utilized for investigating the relationships among various kinds of other measurements: 13 job stressors and exposures, 28 health-related outcomes, 14 work-related outcomes, 19 individual and behavioral factors, and three buffering and non-work factors. For health outcomes, the most often used measurement was depression and anxiety (n = 17). For instance, Tsuboi et al. [51] investigated the association between job stressors and depressive symptoms measured by the center for epidemiologic studies with a depression scale. They compared and categorized female nurses into the most stressful group and the least stressful group and reported a significant difference in depressive symptoms between the two groups. Sakamoto et al. [75] investigated the structural differences among factors for psychological job stress among healthcare workers and reported that job stressors from the BJSQ were positively associated with depression and anxiety measured by the hospital anxiety depression scale. In addition, sleep/insomnia/circadian rhythm (n = 11) were frequently investigated for their association with job stressors. Toyoshima et al. [55] examined interrelationships among sleep reactivity, job-related stress, and subjective cognitive dysfunction and indicated that sleep reactivity significantly influenced subjective cognitive dysfunction directly and indirectly via job stressors and stress responses. Takahashi et al. [46] conducted a one-year longitudinal study to examine how a change in work time control was associated with sleep and health. They indicated that daytime sleepiness was positively associated with quantitative job overload and negatively associated with job control and social support from the BJSQ.

Physiological health outcomes were also tested using the subscales of the BJSQ, such as diabetes, insulin resistance, and blood glucose (n = 4), serum lipid and cholesterols (n = 4), salivary or fingernails cortisol (n = 2), and inflammatory markers (n = 1). For example, Sugito et al. [23] conducted a retrospective study with male workers to investigate the effects of job stressors on the onset of diabetes mellitus defined by HbA1c or using antidiabetic drugs. They indicated that low skill utilization from the BJSQ was associated with the risk of diabetes mellitus onset. Watanabe et al. [101] examined interrelationships between job resources, vigor, exercise habit, and serum lipids including triglyceride, high-density lipoprotein cholesterol, and low-density lipoprotein cholesterol. Multiple-group path analysis indicated that job resources and vigor from the BJSQ were inversely associated with triglyceride and low-density lipoprotein cholesterol and positively associated with high-density lipoprotein cholesterol through exercise habits in both sexes. Nakata et al. [121] investigated associations between job stressors and inflammatory markers including high-sensitive C-reactive protein, interleukin-6, tumor necrosis factor-alpha, monocyte, and leukocyte. The job strain index calculated by dividing quantitative job overload by job control was negatively associated with tumor necrosis factor-alpha.

For work-related outcomes, burnout (n = 7) and presenteeism (n = 7) were often investigated as those associations with PF at work. Saijo et al. [106] investigated the synergistic interaction of job demands, job control, and social support on mental health among local government employees. They indicated significant associations between these stressors from the BJSQ and burnout measured by the Japanese version of the Maslach Burnout Inventory-General Survey. Hayashida et al. [57] assessed the association between the irregularity of mealtimes and presenteeism measured by the Work Limitations Questionnaire. They indicated that the irregularity of mealtimes had a strong effect on presenteeism indirectly through psychological and physical stress responses from the BJSQ.

Coping (n = 10) and sense of coherence (n = 6) were frequently used as individual and behavioral factors. For instance, Shimazu et al. [49] examined the lagged effects of active coping on stress responses to explain the individual differences in the underlying mechanisms behind the association between job stressors and health outcomes. They measured active coping using the Brief Stress for Coping Scale and reported significant interactions of quantitative job overload, job control, and active coping on stress responses from the BJSQ. Urakawa et al. [45] examined the association between a sense of coherence and psychological responses and reported that a sense of coherence was inversely associated with psychological and physical stress responses.

A total of five reports used subscales from the New BJSQ [30,36,81,94,111]. Morimoto et al. [30] investigated the adverse effects of role conflict on the psychological strain among employed family caregivers of people with dementia. They used the subscales of emotional demands and role conflict from the New BJSQ, in addition to the subscales from the BJSQ. They indicated that conflict between caregiving and work was positively associated with psychological strain and its association was moderated by formal support seeking and attentional control. Sakuraya et al. [36] used a three-item scale of workplace social capital and investigated its association with the onset of major depressive episodes through a three-year prospective cohort study. The study indicated that middle-level workplace social capital had the lowest risk of major depressive episodes. Inaba [81] and Inaba and Inoue [111] used multiple subscales from the short version of the New BJSQ and examined their associations with subjective well-being and burnout among female nurses. They indicated that burnout was significantly associated with role conflict, role clarity, and job security [111], and subjective well-being was significantly associated with career development [81]. Toyama and Mauno [94] used the three-item subscale of realization of creativity and reported a significant and positive association with emotional intelligence among eldercare nurses in special nursing homes.

### 3.4. Scoring Methods

Concerning scoring methods, most studies used continuous scores of the subscales (n = 111). Categorization using means, medians, tertiles, and quantiles was also adopted (n = 12). Standardized scores on a five-point scale (n = 7) were calculated based on the distribution of continuous scores in the representative sample [176]. For more practical and easier scoring, a simple scoring method was used (n = 9) [177]. In this method, the respondents were dichotomized into stressed or not stressed, by counting how many items of the BJSQ were scored as undesirable. The definition of “high-stress” employees according to the Japanese NSCP was also used (n = 7) [32]. This definition is conceptualized by the combination of high scores in stress response, high scores in job stressors, and low scores in social support. The predictive validity of the “high-stress” employees for long-term sickness absence at the one-year follow-up was confirmed in a previous study [32].

## 4. Discussion

### 4.1. Main Findings

In the last two decades, over 140 observational studies using the BJSQ and/or the New BJSQ have been published. Since 2015, when the NSCP was launched, large-scale data from more than 60,000 people have been published, as the assessment of psychosocial stress in employees became mandatory. Although not all reports were written in English, more than two-thirds were readable, at least with abstracts that were in English, and more than 100 articles were identifiable by digital object identifiers. Associations were established between a wide variety of factors, including job stressors, health-related outcomes, work-related outcomes, individual and behavioral factors, and buffering factors. The relationship with physical biomarkers was also examined. Although not all studies observed significant associations between factors, and not all study hypotheses were supported, the reported associations were generally reasonable and consistent with existing findings about job stress models. This means that the mechanism that exposure to job stressors evokes deterioration of health- and work-related outcomes and that some of these associations are modified by individual and behavioral factors. Therefore, the BJSQ and the New BJSQ are questionnaires that have made substantial contributions to the research and practice of occupational stress in Japan.

### 4.2. Theoretical Implications

The reasonable associations with validated measurements of health- and work-related outcomes were repeatedly observed in multiple subscales of the BJSQ. In particular, quantitative job overload, job control, supervisor and coworker support, and stress responses often had significant associations with depression and anxiety, quality of life, sleepiness, burnout, sickness absence, and physical biomarkers. These results may reflect the construct validity (concurrent and predictive) of the subscales, while the BJSQ is easy to answer because of the low number of items in each subscale (three at most). The subscales of quantitative job overload, job control, and social support at work can be used as the representative job stressors, referring to the job demands–control model [174,175] as the theoretical background. The subscales of the psychological and physical stress response may also be useful as the indicators of broad symptoms evoked by exposure to stressful PF at work. These subscales may be used as the outcomes of the intervention study. In contrast, compared to the subscales from the BJSQ, those from the New BJSQ were not much considered in the research. More studies are needed to confirm the psychometric validity using the subscales from the New BJSQ.

### 4.3. Practical Implications

Scoring methods are inconsistent among studies, which is partly because these were developed so that the BJSQ can be used for both research and practice. The predictive validity of sickness absence has been confirmed for “high-stress” employees in the NSCP. It is necessary to use the appropriate method according to the purpose of use.

Translation into other languages is the next interest in research and practice. Several studies were conducted in other countries but were not included because the validity of the translated scales could not be verified [171,172,173]. Recently, the BJSQ and the New BJSQ have been translated into English, Chinese, Portuguese, Myanmar, Vietnamese, Spanish, Tagalog, Nepali, Persian, and Indonesian [178,179], and the use of these scales in other countries have already been reported in a peer-reviewed journal [180]. The translated version of the scales can be used as tools to promote not only research on foreign workers in each region of Japan but also job stress research in other countries and international job stress research.

### 4.4. Limitations

There are several limitations to this study. The study quality and risk of bias of the included studies were not assessed because the objective of this study was limited to summarizing published information related to the BJSQ and the New BJSQ. Since most of the included studies were conducted cross-sectionally, the findings from each study could include substantial biases. Further, a body of evidence for the associations between the subscales from the BJSQ and other measurements could not be presented.

## 5. Conclusions

In conclusion, as a comprehensive questionnaire, the BJSQ and the New BJSQ have contributed to the measurement of PF at work and the publication of scientific papers in the occupational health field. The BJSQ can be one of the methodological tools to explore the mechanisms between job stress and several work-related disorders and can provide hints of intervention. Quantitative job overload, job control, and supervisor and coworker support were often used and may have the construct validity as the representative job stressors, referring to the job demands–control model. Regarding practical implication, using the appropriate scoring method according to the usage purpose is important. Prospective, interventional, and multilingual studies are expected to be published to accumulate more comprehensive and high-quality findings in the future.

## Data Availability

Since this study is a systematic review, no individual data is available. The summarized data for this systematic review can be obtained upon request.

## Appendix A

**Table A1:** Summary of the included studies that used the Brief Job Stress Questionnaire.

No.	Author	Study Design	Recruitment	Sample	Subscale Used	Scoring	Other Measurements
1 [23]	Sugito et al. (2021)	Longitudinal	Private companies	6620 male company workers aged 40 years or older who underwent routine annual health checkups and a stress check	Quantitative job overload (3 items) Qualitative job overload (3 items) Physical demands (1 item) Interpersonal conflict (3 items) Poor physical environment (1 item) Job control (3 items) Skill utilization (1 item) Suitable jobs (1 item) Meaningfulness of work (1 item) Vigor (3 items) Anger-irritability (3 items) Fatigue (3 items) Anxiety (3 items) Depression (6 items) Physical stress response (11 items) Supervisor support (3 items) Coworker support (3 items) Support from family and friends (3 items) Job and life satisfaction (2 items)	Standardized scores were calculated on a five-point scale, ranging from 1 (lowest) to 5 (highest).	Development of diabetes mellitus (HbA1c by blood sampling or using antidiabetic drugs)
2 [24]	Takahashi et al. (2020)	Longitudinal	Healthcare centers	6326 male workers who received annual health checkups	Vigor (3 items) Anger-irritability (3 items) Fatigue (3 items) Anxiety (3 items) Depression (6 items) Physical stress response (11 items)	Participants were dichotomized into positive or negative; points 1 and 2 received a score of 0, and points 3 and 4 received a score of 1. An individual was considered positive for depression when they scored at least 1 in each of the depression-related items.	Development of type 2 diabetes (HbA1c by blood sampling or diabetes medication)
3 [25]	Kachi et al. (2020)	Longitudinal	Private companies	9657 workers at a financial service company	Job stressors (17 items) Stress response (29 items) Social support (9 items)	Participants were categorized into high stress or not, as defined by the Stress Check Program in Japan.	Turnover (Human resource records)
4 [26]	Shimazaki et al. (2020)	Longitudinal	Private companies	635 workers of small- to medium-sized enterprises	Vigor (3 items) Anger-irritability (3 items) Fatigue (3 items) Anxiety (3 items) Depression (6 items)	Continuous score	Mental health promotion behaviors (Mental Health Promotion Behavior scale, MHPB)
5 [27]	Wang et al. (2020)	Longitudinal	Private companies	307 full-time and white-collar employees in wide-ranging occupations	Anger-irritability (3 items) Fatigue (3 items) Anxiety (3 items) Depression (6 items) Physical stress response (11 items)	Continuous score	Ethical leadership (Ethical Leadership Scale) Mutual support (Self-developed, validated previously)
6 [28]	Inoue et al. (2019)	Longitudinal	Private companies	14,687 employees in a financial service company	Job satisfaction (1 item)	Participants who answered 1 or 2 were dichotomized into “dissatisfied” and those who answered 3 or 4 into “satisfied” groups.	Long-term sickness absence (Personnel records)
7 [29]	Hino et al. (2019)	Longitudinal	Private companies	922 workers in a manufacturing company in Japan	Depression (6 items)	Continuous score	Overtime work hours (Personnel records)
8 [30]	Morimoto et al. (2019)	Longitudinal	Nursing or welfare facilities	379 employed family caregivers of people with dementia	Quantitative job overload (3 items) Qualitative job overload (3 items) Physical demands (1 item) Interpersonal conflict (3 items) Poor physical environment (3 items) Emotional demands (1 item) Role conflict (1 item)	Continuous score	Psychological strain (Stress Response Scale, SRS)
9 [31]	Ogawa et al. (2018)	Nested case-control study	Public sectors	382 public servants in the Kinki area	Quantitative job overload (3 items) Qualitative job overload (3 items) Physical demands (1 item) Interpersonal conflict (3 items) Poor physical environment (1 item) Job control (3 items) Skill utilization (1 item) Suitable jobs (1 item) Meaningfulness of work (1 item) Vigor (3 items) Anger-irritability (3 items) Fatigue (3 items) Anxiety (3 items) Depression (6 items) Physical stress response (11 items) Supervisor support (3 items) Coworker support (3 items) Support from family and friends (3 items) Job satisfaction (1 item) Satisfaction with family life (1 item)	Continuous score	Long-term sickness absence due to mental disorders (Doctor’s medical certification)
10 [32]	BJSQ et al. (2018)	Longitudinal	Private companies	7356 male and 7362 female employees in a financial service company	Job stressors (17 items) Stress response (29 items) Social support (9 items)	Participants were categorized into high stress or not, as defined by the Stress Check Program in Japan.	Sickness absence (Human resources records)
11 [33]	Fukuda et al. (2018)	Longitudinal	Public sectors	16,032 public servants in the Kinki area	Job stressors (17 items) Stress responses (29 items) Social support (9 items)	Continuous score	Long-term sickness absence due to mental disorders in the same work unit (Medical certification)
12 [34]	Okita et al. (2017)	Longitudinal	Hospitals	42 female novice nurses at Kagoshima University Hospital	Psychological stress response (18 items) Physical stress response (11 items)	Continuous score	Physical examination parameters (Blood sampling) Urinary parameters (Urine sampling) One-year body weight change
13 [35]	Hino et al. (2016)	Longitudinal	Healthcare centers	1815 male workers who underwent health checkups at a healthcare center in the Kanto (east coast) region	Quantitative job overload (3 items) Job control (3 items) Supervisor support (3 items) Coworker support (3 items)	A four-category variable for each psychosocial work characteristic was created (1) stable low, (2) increased, (3) decreased, and (4) stable high group. The demands/control ratio was also calculated.	Insulin resistance (Blood sampling)
14 [36]	Sakuraya et al. (2016)	Longitudinal	Private companies	929 employees from a thinktank company	Workplace social capital (3 items)	Respondents were grouped into tertiles (high, middle, and low). Further, respondents were categorized into tertiles based on the distribution of each item score.	Onset of major depressive episode (World Health Organization’s version of Composite International Diagnostic Interview 3.0, WHO-CIDI 3.0)
15 [37]	Taniguchi et al. (2016)	Longitudinal	Nursing or welfare facilities	543 workers at welfare facilities for the elderly.	Psychological stress response (18 items) Physical stress response (11 items)	The participants were dichotomized into high and low stress groups. For psychological stress response, >13 and >12 indicated a high score in men and women, respectively. For physical stress response, >4 or >5 indicated a high score in men or women, respectively.	Workplace bullying (Japanese version of the Negative Acts Questionnaire, NAQ)
16 [38]	Watanabe et al. (2016)	Longitudinal	Private companies	126 employees from 15 worksites	Psychological stress response (18 items)	Continuous score	Physical activity (Japanese version of the International Physical Activity Questionnaire, IPAQ)
17 [39]	Endo et al. (2015)	Longitudinal	Private companies	540 employees from one of the biggest telecommunication companies	Quantitative job overload (3 items) Job control (3 items)	Based on the means of “organizational job demands” or “organizational job control” scores, the departments were dichotomized into two groups (high/low).	Recurrent sickness absence due to depression (Psychiatric certification)
18 [40]	Shimazu et al. (2015)	Longitudinal	Private companies	1196 employees in an industrial machinery company	Psychological stress response (18 items) Physical stress response (11 items) Job satisfaction (1 item) Satisfaction with family life (1 item)	Continuous score	Work engagement (Utrecht Work Engagement Scale, UWES) Workaholism (Dutch Workaholism Scale, DUWAS)
19 [41]	Matsudaira et al. (2014)	Longitudinal	Existing cohorts	3811 workers from a prospective cohort of the “The Japan epidemiological research of Occupation-related Back pain (JOB)” study.	Quantitative job overload (3 items) Qualitative job overload (3 items) Physical demands (1 item) Interpersonal conflict (3 items) Poor physical environment (1 item) Job control (3 items) Skill utilization (1 item) Suitable jobs (1 item) Vigor (3 items) Anger-irritability (3 items) Fatigue (3 items) Anxiety (3 items) Depression (6 items) Physical stress response (11 items) Supervisor support (3 items) Coworker support (3 items) Support from family and friends (3 items) Job satisfaction (1 item) Satisfaction with family life (1 item)	For each factor, standardized scores were developed on a five-point scale ranging from 1 (lowest) to 5 (highest) based on a sample of more than 10,000 Japanese workers. The five original responses were reclassified into “not feeling stressed,” where low, slightly low, and moderate were combined, and “feeling stressed,” where slightly high and high were combined.	Low back pain (Von Korff’s grading method)
20 [42]	Wada et al. (2013)	Longitudinal	Web surveys	1810 participants aged 20–70 years from a marketing survey	Stress responses (29 items)	The participants were divided into quartiles according to the total stress response score at baseline.	Sick leave due to depression (Medical certificates)
21 [43]	Demerouti et al. (2013)	Longitudinal	Existing cohorts	471 Japanese employees with young children from the Tokyo Work–family INterface (TWIN) study	Supervisor support (3 items)	Continuous score	Work–self facilitation (Four items based on the Survey Work–home Interference Nijmegen, SWING)
22 [44]	Okuno et al. (2013)	Longitudinal	Hospitals	105 nurses in from a hospital	Quantitative job overload (3 items) Qualitative job overload (3 items) Physical demands (1 item) Interpersonal conflict (3 items) Poor physical environment (1 item) Job control (3 items) Skill utilization (1 item) Suitable jobs (1 item) Meaningfulness of work (1 item) Supervisor support (3 items) Coworker support (3 items)	Continuous score	Post-traumatic growth (Japanese version of Posttraumatic Growth Inventory, PTGI-J)
23 [45]	Urakawa et al. (2012)	Longitudinal	Private companies	299 employees in small enterprises	Vigor (3 items) Anger-irritability (3 items) Fatigue (3 items) Anxiety (3 items) Depression (6 items) Physical stress response (11 items)	Continuous score	Sense of coherence (Japanese version of Sense of Coherence Scale, SOC)
24 [46]	Takahashi et al. (2012)	Longitudinal	Web surveys	2382 daytime workers selected randomly from a market research panel	Quantitative job overload (3 items) Job control (3 items) Supervisor and coworker support (6 items)	Continuous score	Fatigue (An 11-item scale from the Checklist for Accumulated Fatigue due to Overwork) Depressive symptoms (Center for Epidemiologic Studies for Depression scale, CES-D) Work time control (A five-item scale from Takahashi et al. (2011)) Daytime sleepiness (Epworth Sleepiness Scale)
25 [47]	Sugimura and Thériault (2010)	Cross-sectional Longitudinal	Private companies	1157 male employees in an information technology company	Supervisor support (3 items)	Cross-sectional survey: supervisor support score was categorized into four groups for every quartile score. Longitudinal survey: supervisor support scores of each survey period were dichotomized based on the median score to create four dual categories that take into account the changes in supervisor support between the survey periods (i.e., low [T1]–low [T2], low [T1]–high [T2], high [T1]–low [T2], and high [T1]–high [T2]). Continuous score	Work ability (Work Ability Index, WAI)
26 [48]	Shimazu and de Jonge (2009)	Longitudinal	Private companies	211 employees in a construction machinery company	Psychological stress response (18 items) Physical stress response (11 items)	Continuous score	Effort–reward imbalance (Japanese version of Effort–Reward Imbalance Questionnaire, ERI-Q)
27 [49]	Shimazu et al. (2008)	Longitudinal	Private companies	193 employees working in a construction machinery company	Quantitative job overload (3 items) Job control (3 items) Psychological stress response (18 items) Physical stress response (11 items)	Continuous score	Active coping (Brief Stress for Coping Scale, BSCP)
28 [50]	Shimazu and Schaufeli (2007)	Longitudinal	Private companies	488 male employees in a construction machinery company	Job stressors (17 items) Stress responses (29 items)	Continuous score	Coping (Brief Stress for Coping Scale, BSCP) Job performance (World Health Organization Health and Work Performance Questionnaire, WHO-HPQ)
29 [51]	Tsuboi et al. (2006)	Longitudinal	Hospitals	33 female nurses working in Fujita Health University Hospital	Job stressors (17 items)	Those who scored more “1”s were placed in a high job stress group and those who scored more “5”s were placed in a low job stress group, according to the manual of the BJSQ.	Cholesterols Lipid peroxidation antioxidants in the plasma (Blood sampling) Depressive symptoms (Center for Epidemiologic Studies for Depression scale, CES-D)
30 [52]	Hirokawa et al. (2022) (Epub was published in 2021)	Cross-sectional	Healthcare centers	766 healthy workers enrolled in mental health checkups	Quantitative job overload (3 items) Qualitative job overload (3 items) Physical demands (1 item) Job control (3 items) Skill utilization (1 item) Suitable jobs (1 item) Supervisor support (3 items) Coworker support (3 items) Job satisfaction (1 item)	Continuous score	Salivary cortisol (Saliva sampling)
31 [53]	Takaesu et al. (2021)	Cross-sectional	Private companies	4645 office workers from 29 companies	Job stressors (17 items) Stress response (29 items) Social support (9 items)	Participants were categorized into high stress or not, defined by the Stress Check Program in Japan.	Sleep duration (Self-reported)
32 [54]	Hidaka et al. (2021)	Cross-sectional	Web surveys	1986 workers from the Internet survey	Quantitative job overload (3 items) Job control (3 items) Supervisor support (3 items) Coworker support (3 items)	Job strain was calculated by dividing quantitative job overload by job control. Social support was used in continuous score.	Health-related quality of life (EQ-5D-5L)
33 [55]	Toyoshima et al. (2021)	Cross-sectional	Convenience sample of faculty staff members or alumni of universities	536 workers from the recruitment that performed through the word of mouth, using poster at the Tokyo Medical University	Job stressors (17 items) Stress response (29 items) Social support (9 items) Job and life satisfaction (2 items)	Continuous score	Sleep reactivity (The Ford Insomnia Response to Stress Test, FIRST) Cognitive dysfunction (The Cognitive Complaints in Bipolar Disorder Rating Assessment, COBRA)
34 [56]	Adachi et al. (2021)	Cross-sectional	Healthcare centers	2739 university workers who underwent an annual health checkup at the Health and Counseling Center, Osaka University	Job stressors (17 items) Stress response (29 items) Social support (9 items)	Continuous score	Sleep duration (Self-reported)
35 [57]	Hayashida et al. (2021)	Cross-sectional	Private companies	2905 workers from 17 offices at companies	Stress response (29 items)	Continuous score	Presenteeism (Work Limitations Questionnaire, WLQ) Sleep quality (Pittsburgh Sleep Quality Index, PSQI)
36 [58]	Ôga and Chiba (2021)	Cross-sectional	Hospitals	765 nurses from eight hospitals that have 100 or more beds in Tohoku, Japan	Vigor (3 items) Anger-irritability (3 items) Fatigue (3 items) Anxiety (3 items) Depression (6 items) Physical stress response (11 items) Coworker support (3 items)	Continuous score	Humor expression (Humor Expression Scale)
37 [59]	Adachi (2021)	Cross-sectional	Private companies	114 workers in a manufacturing company	Quantitative job overload (3 items) Qualitative job overload (3 items) Physical demands (1 item) Interpersonal conflict (3 items) Poor physical environment (1 item) Job control (3 items) Skill utilization (1 item) Suitable jobs (1 item) Meaningfulness of work (1 item) Vigor (3 items) Anger-irritability (3 items) Fatigue (3 items) Anxiety (3 items) Depression (6 items) Physical stress response (11 items) Supervisor support (3 items) Coworker support (3 items) Support from family and friends (3 items) Job and life satisfaction (2 items)	Continuous score	Shift work (Self-reported)
38 [60]	Ooka et al. (2021)	Cross-sectional	Healthcare centers	69,805 workers in 117 companies that conducted the national Stress Check Program through Public Health Research Center	Job stressors (17 items) Stress response (29 items) Social support (9 items)	Participants were categorized into high stress or not, as defined by the Stress Check Program in Japan.	Shift work (Self-reported) Overtime work hours (Self-reported)
39 [61]	Terada and Nagamine (2020)	Cross-sectional	Public sectors	326 male workers of the Japan Ground Self-Defense Force	Job stressors (17 items) Stress response (29 items)	Continuous score	Coping (Tri-Axial Coping Scale, TAC-24) Resilience (Japanese Short version of Resilience Competency Scale, RCS-JS) Hardiness (Validated scale)
40 [62]	Sameshima et al. (2020)	Cross-sectional	Convenience sample of faculty staff members or alumni of universities	528 nonclinical workers recruited by convenience sampling through our acquaintances at Tokyo Medical University	Job stressors (17 items) Stress response (29 items)	Continuous score	Parenting quality (Parental Bonding Instrument) Resilience (Connor-Davidson Resilience Scale)
41 [63]	Shimura et al. (2020)	Cross-sectional	Private companies	5640 workers from 29 companies in Tokyo	Job stressors (17 items) Social support (9 items)	Continuous score	Sleep schedule (Self-reported) Sleep quality (Pittsburgh Sleep Quality Index, PSQI)
42 [64]	Furuichi et al. (2020)	Cross-sectional	Private companies	2899 workers from 17 companies in Tokyo, Japan	Job stressors (17 items) Stress response (29 items) Social support (9 items)	Continuous score	Presenteeism (Work Limitation Questionnaire, WLQ) Sleep quality (Pittsburgh Sleep Quality Index, PSQI)
43 [65]	Miyama et al. (2020)	Cross-sectional	Convenience sample of faculty staff members or alumni of universities	535 nonclinical workers recruited by convenience sampling through our acquaintances at Tokyo Medical University	Job stressors (17 items) Stress response (29 items) Social support (9 items) Job and life satisfaction (2 items)	Continuous score	Diurnal type (Diurnal Type Scale, DTS) Sleep quality (Pittsburgh Sleep Quality Index, PSQI)
44 [66]	Seki et al. (2020)	Cross-sectional	Convenience sample of faculty staff members or alumni of universities	528 workers recruited by convenience sampling through our acquaintances at Tokyo Medical University	Job stressors (17 items) Stress response (29 items)	Continuous score	Parenting quality (Parental Bonding Instrument) Neuroticism (Eysenck Personality Questionnaire-Revised, EPQ-R)
45 [67]	Kikuchi et al. (2020)	Cross-sectional	Healthcare centers	59,021 workers in 117 companies that implemented the national Stress Check Program	Vigor (3 items) Anger-irritability (3 items) Fatigue (3 items) Anxiety (3 items) Depression (6 items) Physical stress response (11 items)	Continuous score	Overtime work hours (Self-reported)
46 [68]	Taya et al. (2020)	Cross-sectional	Private companies	2905 workers from 17 worksites in Tokyo, Japan	Job stressors (17 items) Stress response (29 items) Social support (9 items)	Continuous score	Presenteeism (Work Limitation Questionnaire, WLQ)
47 [69]	Hayasaki et al. (2020)	Cross-sectional	Hospitals	284 nurses from 12 wards in a hospital	Job stressors (17 items) Stress response (29 items) Social support (9 items)	The participants were dichotomized into high and low groups. For each subscale, high stress was defined by counting the number of non-desirable answers.	Ethical behaviors of nurses (Validated scale)
48 [70]	Saito et al. (2020)	Cross-sectional	Nursing or welfare facilities	49 teachers who have been employed for 5–10 years by nursing schools in an area in Japan	Stress response (29 items)	Continuous score	Self-efficacy (Generalized Self-Efficacy Scale, GSES)
49 [71]	Okamoto et al. (2020)	Cross-sectional	Nursing or welfare facilities	616 healthcare workers from 217 welfare facilities for the disabled in the Chugoku area, Japan	Supervisor support (3 items) Coworker support (3 items)	Respondents were grouped into tertiles (high, middle, and low).	Inappropriate care (Validated scale)
50 [72]	Nagata et al. (2019)	Cross-sectional	Private companies	2693 employees at a pharmaceutical company	Quantitative job overload (3 items) Qualitative job overload (3 items) Job control (3 items) Supervisor support (3 items) Coworker support (3 items)	Continuous score	Psychological distress (K6) Work engagement (Utrecht Work Engagement Scale, UWES)
51 [73]	Okawa et al. (2019)	Cross-sectional	Private companies	103 employees at 17 corporations in Kanagawa Prefecture, Japan	Job stressors (17 items) Stress response (29 items) Social support (9 items)	Participants were defined as high stress or not based on the Stress Check Program in Japan.	Autonomic nervous activity (Electrocardiography, photoplethysmography)
52 [74]	Maeda et al. (2019)	Cross-sectional	Web surveys	2000 female workers from an online research panel living with a partner	Quantitative job overload (3 items) Job control (3 items) Supervisor support (3 items) Coworker support (3 items)	Continuous score	Psychological health (mental health and vitality) (SF-36)
53 [75]	Sakamoto et al. (2019)	Cross-sectional	Hospitals	205 healthcare workers from a core hospital	Quantitative job overload (3 items) Qualitative job overload (3 items) Physical demands (1 item) Interpersonal conflict (3 items) Poor physical environment (1 item) Job control (3 items) Skill utilization (1 item) Suitable jobs (1 item) Meaningfulness of work (1 item) Vigor (3 items) Anger-irritability (3 items) Fatigue (3 items) Anxiety (3 items) Depression (6 items) Physical stress response (11 items) Supervisor support (3 items) Coworker support (3 items) Support from family and friends (3 items) Job and life satisfaction (2 items)	Continuous score	Depression and anxiety (Hospital Anxiety Depression Scale, HADS) Chronic pain (Pain Catastrophizing Scale, PCS)
54 [76]	Matsumoto and Yoshioka (2019)	Cross-sectional	Hospitals	577 psychiatric nurses working at 13 psychiatric hospitals with more than 150 beds in the Chugoku area	Job stressors (17 items) Supervisor support (3 items) Coworker support (3 items) Job satisfaction (1 item)	Continuous score	Negative Feeling toward patient (Negative Feeling toward Patient Frequency scale) Emotional, evaluative, informative, and instrumental support (Support-in-workplace scale)
55 [77]	Kurebayashi (2019)	Cross-sectional	Hospitals	271 general and 316 psychiatric nurses from seven hospitals in Japan	Quantitative job overload (3 items) Qualitative job overload (3 items) Physical demands (1 item) Interpersonal conflict (3 items) Poor physical environment (1 item) Job control (3 items) Skill utilization (1 item) Suitable jobs (1 item) Meaningfulness of work (1 item) Vigor (3 items) Anger-irritability (3 items) Fatigue (3 items) Anxiety (3 items) Depression (6 items) Physical stress response (11 items) Supervisor support (3 items) Coworker support (3 items) Support from family and friends (3 items) Job and life satisfaction (2 items)	Continuous score	Nursing skills (Self-Evaluation Scale of Oriented Problem Solving Behavior, OPSN)
56 [78]	Watanabe and Yamauchi (2019)	Cross-sectional	Hospitals	1075 full-time nurses working in four hospitals in Japan	Fatigue (3 items)	Continuous score	Motivation for overtime work (Self-developed, validated in the study)
57 [79]	Fukunaga et al. (2019)	Cross-sectional	Nursing or welfare facilities	312 teachers who intended to change occupations from nursing schools in eight prefectures	Quantitative job overload (3 items) Job control (3 items) Fatigue (3 items) Anxiety (3 items) Depression (3 items) Physical stress response (2 items) Supervisor support (3 items) Coworker support (3 items)	Continuous score	Identity of nurses and nursing teachers (Validated scale)
58 [80]	Yoneyama et al. (2019)	Cross-sectional	Hospitals	215 female nurses from advanced treatment hospitals in Kanto area, Japan	Quantitative job overload (3 items) Qualitative job overload (3 items) Physical demands (1 item) Interpersonal conflict (3 items) Poor physical environment (1 item) Job control (3 items) Skill utilization (1 item) Suitable jobs (1 item) Meaningfulness of work (1 item) Vigor (3 items) Anger-irritability (3 items) Fatigue (3 items) Anxiety (3 items) Depression (6 items) Physical stress response (11 items) Supervisor support (3 items) Coworker support (3 items) Support from family and friends (3 items) Job and life satisfaction (2 items)	Continuous score	Recognition of the organization (Validated scale)
59 [81]	Inaba (2018)	Cross-sectional	Hospitals	318 female nurses working in a private hospital for one or more years	Quantitative job overload (3 items) Qualitative job overload (3 items) Physical demands (1 item) Job control (3 items) Interpersonal conflict (3 items) Poor physical environment (1 item) Skill utilization (1 item) Suitable jobs (1 item) Meaningfulness of work (1 item) Emotional demands (1 item) Role conflict (1 item) Work–self balance (negative) (1 item) Role clarity (1 item) Career opportunity (1 item) Supervisor support (3 items) Coworker support (3 items) Support from family and friends (3 items) Monetary/status reward (1 item) Esteem reward (1 item) Job security (1 item) Leadership (1 item) Interactional justice (1 item) Workplace where people compliment each other (1 item) Workplace where mistakes are acceptable (1 item) Collective efficacy (1 item) Trust with management (1 item) Preparedness for change (1 item) Respect for individuals (1 item) Fair personnel evaluation (1 item) Diversity (1 item) Career development (1 item) Work–self balance (positive) (1 item) Vigor (3 items) Anger-irritability (3 items) Fatigue (3 items) Anxiety (3 items) Depression (6 items) Physical stress response (11 items) Job satisfaction (1 item) Satisfaction with family life (1 item) Workplace harassment (1 item) Workplace social capital (1 item)	Continuous score	Subjective well-being (Single item)
60 [82]	Sato (2018)	Cross-sectional	Private companies	109 workers from four worksites	Quantitative job overload (3 items) Qualitative job overload (3 items) Physical demands (1 item) Interpersonal conflict (3 items) Poor physical environment (1 item) Job control (3 items) Skill utilization (1 item) Suitable jobs (1 item) Meaningfulness of work (1 item) Vigor (3 items) Anger-irritability (3 items) Fatigue (3 items) Anxiety (3 items) Depression (6 items) Physical stress response (11 items) Supervisor support (3 items) Coworker support (3 items) Support from family and friends (3 items)	Continuous score Participants were defined as high stress or not based on the Stress Check Program in Japan.	Risk of periodontal disease (Saliva sampling)
61 [83]	Horie et al. (2018)	Cross-sectional	Nursing or welfare facilities	18 faculty staff members from care worker schools	Quantitative job overload (3 items) Qualitative job overload (3 items) Physical demands (1 item) Interpersonal conflict (3 items) Poor physical environment (1 item) Job control (3 items) Skill utilization (1 item) Suitable jobs (1 item) Meaningfulness of work (1 item) Vigor (3 items) Anger-irritability (3 items) Fatigue (3 items) Anxiety (3 items) Depression (6 items) Physical stress response (11 items)	Continuous score	Burnout (Maslach Burnout Inventory, MBI)
62 [84]	Nakamura and Mizukami (2018)	Cross-sectional	Nursing or welfare facilities	657 healthcare workers at nine elderly nursing homes	Vigor (3 items) Anger-irritability (3 items) Fatigue (3 items) Anxiety (3 items) Depression (6 items) Physical stress response (11 items)	Continuous score	Work value (Work value scale) Assertive mind (Assertive Mind Scale) Self-efficacy (Generalized Self-efficacy Scale, GSES) Sense of coherence (Japanese sense of coherence scale: SOC-13) Communication skills (ENDCOREs) Working behaviors (Working behavior scale) Problem solution ability (Problem solution ability scale)
63 [85]	Enoki et al. (2018)	Cross-sectional	Private companies	664 workers from a call center	Quantitative job overload (3 items) Job control (3 items) Supervisor support (3 items) Coworker support (3 items)	Continuous score	Sleeping time (Self-reported)
64 [86]	Okada et al. (2018)	Cross-sectional	Hospitals	108 female nurses who are wives or mothers from two general hospitals in Fukuoka Prefecture, Japan	Job stressors (17 items) Stress response (29 items) Social support (9 items)	Continuous score	Mental health (28-item General Health Questionnaire, GHQ-28)
65 [87]	Adachi and Inaba (2018)	Cross-sectional	Private companies	368 workers from a single worksite	Vigor (3 items) Anger-irritability (3 items) Fatigue (3 items) Anxiety (3 items) Depression (6 items) Physical stress response (11 items)	Continuous score	Depression (Center for Epidemiologic Studies for Depression scale, CES-D)
66 [88]	Sakamoto et al. (2018)	Cross-sectional	Hospitals	38 workers of the rehabilitation department of a core hospital	Quantitative job overload (3 items) Qualitative job overload (3 items) Physical demands (1 item) Interpersonal conflict (3 items) Poor physical environment (1 item) Job control (3 items) Skill utilization (1 item) Suitable jobs (1 item) Meaningfulness of work (1 item) Vigor (3 items) Anger-irritability (3 items) Fatigue (3 items) Anxiety (3 items) Depression (6 items) Physical stress response (11 items)	Continuous score	Pain catastrophizing (The Pain Catastrophizing Scale, PCS) Depression and anxiety (Hospital Anxiety Depression Scale, HADS)
67 [89]	Yada et al. (2017)	Cross-sectional	Hospitals	68 psychiatric assistant nurses and 140 psychiatric registered nurses from six psychiatric hospitals.	Vigor (3 items) Anger-irritability (3 items) Fatigue (3 items) Anxiety (3 items) Depression (6 items) Physical stress response (11 items)	Continuous score	Psychiatric nurse job stressor (Psychiatric Nurse Job Stressor Scale, PNJSS)
68 [90]	Enoki et al. (2017)	Cross-sectional	Private companies	538 employees from a call center	Quantitative job overload (3 items) Job control (3 items) Supervisor support (3 items) Coworker support (3 items)	Continuous score	Electrocardiography (EDG-9130 electrocardiograph)
69 [91]	Tsutsumi et al. (2017)	Cross-sectional	Web surveys	1650 workers via an online survey	Job stressors (17 items) Stress response (29 items) Social support (9 items)	Participants were defined as high stress or not based on the Stress Check Program in Japan.	Psychological distress (K6 scale)
70 [92]	Saijo et al. (2017)	Cross-sectional	Public sectors	2535 employees in local government, Asahikawa city, Hokkaido	Quantitative job overload (3 items) Job control (3 items) Supervisor support (3 items) Coworker support (3 items) Support from family and friends (3 items)	Continuous score	Work impairment Work output (13-item Stanford Presenteeism Scale, SPS-13)
71 [93]	Sakuraya et al. (2017)	Cross-sectional	Private companies	894 employees from a manufacturing company	Psychological stress response, except vigor (15 items)	Continuous score	Job crafting (Japanese version of the Job Crafting Questionnaire)
72 [94]	Toyama and Mauno (2017)	Cross-sectional	Nursing or welfare facilities	489 eldercare nurses in special nursing homes	Social support (9 items) Realization of creativity (3 items)	Continuous score	Emotional intelligence (Emotional Intelligence Scale, EQS)
73 [95]	Izawa et al. (2017)	Cross-sectional	Hospitals	123 middle-aged workers from hospitals and research institutes in Kanagawa prefecture	Quantitative job overload (3 items) Job control (3 items)	The job strain index was calculated by dividing job demands by job control.	Cortisol level in fingernails (Fingernail sampling)
74 [96]	Watanabe et al. (2017)	Cross-sectional	Existing cohorts	2502 parents (1251 couples) from the Tokyo Work–Family INterface (TWIN) study	Fatigue (3 items)	Continuous score	Job demands (Four items from Furda, 1995) Family demands (Five items from Peeters, 2005)
75 [97]	Yoshimoto et al. (2017)	Cross-sectional	Hospitals	203 workers from a single hospital	Quantitative job overload (3 items) Job control (3 items) Interpersonal conflict (3 items) Supervisor support (3 items) Coworker support (3 items) Support from family and friends (3 items) Job satisfaction (1 item)	Standardized scores were developed with a five-point scale based on a representative sample of Japanese workers. In addition, the participants were dichotomized into “not stressed” and “stressed” groups.	Low back pain (Von Korff’s grading method)
76 [98]	Sakagami (2016)	Cross-sectional	Convenience sample of faculty staff members or alumni of universities	112 male researchers from two academic institutions	Qualitative job overload (3 items)	Continuous score	Ego aptitude (Tokyo University Egogram Version II, TEG-II)
77 [99]	Yamada et al. (2016)	Cross-sectional	Existing cohorts	1764 workers from a pain-associated cross-sectional epidemiological study	Supervisor support (3 items) Coworker support (3 items) Job satisfaction (1 item)	Social support was used in quartiles of scores. Job satisfaction was classified into four categories: dissatisfied, somewhat dissatisfied, relatively satisfied, or satisfied.	Chronic pain (11-point Numeric Rating Scale, NRS) Health-related quality of life (Euro Quality of Life, EQ-5D)
78 [100]	Otsuka et al. (2016)	Cross-sectional	Private companies	42,499 workers from 61 organizations	Quantitative job overload (3 items) Qualitative job overload (3 items) Physical demands (1 item) Job control (3 items) Interpersonal conflict (3 items) Poor physical environment (1 item) Suitable jobs (1 item) Meaningfulness of work (1 item) Supervisor support (3 items) Coworker support (3 items)	Participants were divided by each mean subscale score.	Suicidal ideation (Single item)
79 [101]	Watanabe et al. (2016)	Cross-sectional	Private companies	4543 employees from a beverage manufacturing company	Job control (3 items) Skill utilization (1 item) Suitable jobs (1 item) Meaningfulness of work (1 item) Vigor (3 items)	Continuous score	Serum lipids (Blood sampling)
80 [102]	Adachi and Inaba (2016)	Cross-sectional	Private companies	368 workers from a single worksite	Vigor (3 items) Anger-irritability (3 items) Fatigue (3 items) Anxiety (3 items) Depression (6 items) Physical stress response (11 items)	Continuous score	Depression (K10 scale)
81 [103]	Kawahito (2016)	Cross-sectional	Private companies	34 middle-aged workers from a manufacturing company	Depression (6 items)	Continuous score	Self-complexity (Trait sort test)
82 [104]	Maruya and Tanaka (2016)	Cross-sectional	Nursing or welfare facilities	29 care workers in a facility for persons with intellectual disabilities	Quantitative job overload (3 items) Qualitative job overload (3 items) Physical demands (1 item) Interpersonal conflict (3 items) Poor physical environment (1 item) Job control (3 items) Skill utilization (1 item) Suitable jobs (1 item) Meaningfulness of work (1 item) Vigor (3 items) Anger-irritability (3 items) Fatigue (3 items) Anxiety (3 items) Depression (6 items) Physical stress response (11 items) Supervisor support (3 items) Coworker support (3 items) Support from family and friends (3 items) Job and life satisfaction (2 items)	Continuous score	Quality of life (26-item World Health Organization Quality of Life Questionnaire, WHO QOL26)
83 [105]	Fujita et al. (2016)	Cross-sectional	Private companies	440 workers from 35 units of a health care corporation	Quantitative job overload (3 items) Job control (3 items) Supervisor and coworker support (6 items)	Continuous score	Work engagement (Utrecht Work Engagement Scale, UWES)
84 [106]	Saijo et al. (2015)	Cross-sectional	Public sectors	2121 employees in the local government of Asahikawa city, Hokkaido	Job demands (3 items) Job control (3 items) Supervisor support (3 items) Coworker support (3 items)	Scores were dichotomized based on the median values.	Depression (Japanese version of Patient Health Questionnaire, PHQ-9) Burnout (Japanese version of Maslach Burnout Inventory-General Survey, MBI-GS) Insomnia (Athens Insomnia Scale, AIS)
85 [107]	Morimoto et al. (2015)	Cross-sectional	Hospitals	189 healthcare professionals from three general hospitals.	Psychological stress response (18 items)	Continuous score	Coping orientation Appraisal of coping acceptability (Coping Scale for Task stressors and Job evaluation stressors, CSTJ; Coping Scale for Interpersonal stressors, CSI)
86 [108]	Kagata et al. (2015)	Cross-sectional	Hospitals	306 female hospital ward nurses in the Kanto region	Psychological stress response except for vigor (15 items) Physical stress response (11 items)	Continuous score	Emotional labor (Emotional Labor Inventory for Nurses, ELIN) Work engagement (Utrecht Work Engagement Scale, UWES)
87 [109]	Lee et al. (2015)	Cross-sectional	Private companies	276 workers (126 high-skilled foreign workers and 150 Japanese workers)	Quantitative job overload (3 items) Qualitative job overload (3 items) Interpersonal conflict (3 items) Supervisor support (3 items) Coworker support (3 items) Support from family and friends (3 items) Job satisfaction (1 item) Satisfaction with family life (1 item)	Continuous score	Anxiety Depression Physical complaints (Mutual Intercultural Relations in Plural Societies Questionnaire, MIRIPSQ)
88 [110]	Kato et al. (2015)	Cross-sectional	Hospitals	837 nurses from 5 general hospitals in Hokkaido	Quantitative job overload (3 items) Qualitative job overload (3 items) Physical demands (1 item) Interpersonal conflict (3 items) Poor physical environment (1 item) Job control (3 items) Skill utilization (1 item) Suitable jobs (1 item) Meaningfulness of work (1 item) Vigor (3 items) Anger-irritability (3 items) Fatigue (3 items) Anxiety (3 items) Depression (6 items) Physical stress response (11 items) Supervisor support (3 items) Coworker support (3 items) Support from family and friends (3 items) Job and life satisfaction (2 items)	Continuous score	Excessive daytime sleepiness (Japanese version of the Epworth Sleepiness Scale, JESS)
89 [111]	Inaba and Inoue (2015)	Cross-sectional	Hospitals	195 female nurses working in a general hospital for one or more years	Quantitative job overload (3 items) Qualitative job overload (3 items) Physical demands (1 item) Job control (3 items) Interpersonal conflict (3 items) Poor physical environment (1 item) Skill utilization (1 item) Suitable jobs (1 item) Meaningfulness of work (1 item) Emotional demands (1 item) Role conflict (1 item) Work–self balance (negative) (1 item) Role clarity (1 item) Career opportunity (1 item) Supervisor support (3 items) Coworker support (3 items) Support from family and friends (3 items) Monetary/status reward (1 item) Esteem reward (1 item) Job security (1 item) Leadership (1 item) Interactional justice (1 item) Workplace where people compliment each other (1 item) Workplace where mistakes are acceptable (1 item) Collective efficacy (1 item) Trust with management (1 item) Preparedness for change (1 item) Respect for individuals (1 item) Fair personnel evaluation (1 item) Diversity (1 item) Career development (1 item) Work–self balance (positive) (1 item) Vigor (3 items) Anger-irritability (3 items) Fatigue (3 items) Anxiety (3 items) Depression (6 items) Physical stress response (11 items) Job satisfaction (1 item) Satisfaction with family life (1 item) Workplace harassment (1 item) Workplace social capital (1 item) Work engagement (1 item)	Continuous Score	Burnout (Pine’s burnout scale)
90 [112]	Nakamura and Mizukami (2015)	Cross-sectional	Nursing or welfare facilities	108 healthcare workers at 31 nursing home	Vigor (3 items) Anger-irritability (3 items) Fatigue (3 items) Anxiety (3 items) Depression (6 items) Physical stress response (11 items)	Continuous score	Sense of coherence (Japanese sense of coherence scale: SOC-13)
91 [113]	Igarashi and Iijima (2015)	Cross-sectional	Private companies	99 female workers at five small–middle-sized companies	Quantitative job overload (3 items) Qualitative job overload (3 items) Physical demands (1 item) Interpersonal conflict (3 items) Poor physical environment (1 item) Job control (3 items) Skill utilization (1 item) Suitable jobs (1 item) Meaningfulness of work (1 item) Vigor (3 items) Anger-irritability (3 items) Fatigue (3 items) Anxiety (3 items) Depression (6 items) Physical stress response (11 items) Supervisor support (3 items) Coworker support (3 items) Support from family and friends (3 items) Job and life satisfaction (2 items)	Continuous score	Quality of life (MOS 36-Item Short-Form Health Survey version2: SF-36)
92 [114]	Ohta et al. (2015)	Cross-sectional	Private companies	1558 workers from an information technology company	Quantitative job overload (3 items) Job control (3 items) Supervisor and coworker support (6 items)	Job stress scores were calculated by dividing job demand by job control. Social support was used as continuous scores.	Mental health (28-item General Health Questionnaire, GHQ-28)
93 [115]	Saijo et al. (2014)	Cross-sectional	Convenience sample of faculty staff members or alumni of universities	494 physicians from the entire alumni population of Asahikawa Medical University	Quantitative job overload (3 items) Job control (3 items) Supervisor support (3 items) Coworker support (3 items)	Job demands and job control scores were each dichotomized at a median value, and job strain was categorized. Social support was used in continuous score.	Depressive symptoms (Japanese version of Patient Health Questionnaire, PHQ-9) Burnout (Japanese version of Maslach Burnout Inventory-General Survey, MBI-GS)
94 [116]	Horita and Otsuka (2014)	Cross-sectional	Private companies	200 workers from three manufacturing companies	Quantitative job overload (3 items) Qualitative job overload (3 items) Supervisor support (3 items) Coworker support (3 items) Vigor (3 items) Anger-irritability (3 items) Fatigue (3 items) Anxiety (3 items) Depression (6 items)	Continuous score	Interpersonal helping behavior (Japanese version of Organizational Citizenship Behavior scale)
95 [117]	Matsuzaki et al. (2014)	Cross-sectional	Hospitals	1169 female registered nurses from 26 public hospitals and two private hospitals	Quantitative job overload (3 items) Qualitative job overload (3 items) Physical demands (1 item) Interpersonal conflict (3 items) Poor physical environment (1 items) Job control (3 items) Skill utilization (1 item) Suitable jobs (1 item) Job satisfaction (1 item)	Continuous score	Menopausal symptoms (Greene’s Climacteric Scale)
96 [118]	Yoshida et al. (2014)	Cross-sectional	Hospitals	102 male and female nurses from a university hospital	Stress response (29 items)	Standardized scores were developed on a five-point scale based on a representative sample of Japanese workers.	Sense of coherence (Japanese sense of coherence scale: SOC-13)
97 [119]	Yada et al. (2014)	Cross-sectional	Hospitals	244 psychiatric male and female nurses from six psychiatric hospitals	Vigor (3 items) Anger-irritability (3 items) Fatigue (3 items) Anxiety (3 items) Depression (6 items) Physical stress response (11 items)	Continuous score	Psychiatric nurse job stressor (Psychiatric Nurse Job Stressor Scale, PNJSS)
98 [120]	Kikuchi et al. (2014)	Cross-sectional	Hospitals	386 nurses from a general hospital	Quantitative job overload (3 items) Qualitative job overload (3 items) Physical demands (1 item) Interpersonal conflict (3 items) Poor physical environment (1 item) Job control (3 items) Skill utilization (1 item) Suitable jobs (1 item) Meaningfulness of work (1 item) Supervisor support (3 items) Coworker support (3 items) Support from family and friends (3 items) Job and life satisfaction (2 items)	Continuous score	Depression (K6 scale) Sense of coherence (Japanese sense of coherence scale: SOC-13)
99 [121]	Nakata et al. (2014)	Cross-sectional	Private companies	137 male white-collar workers from a trading company	Quantitative job overload (3 items) Job control (3 items)	The job strain index was calculated by dividing job demands by job control.	Inflammatory markers(Blood sampling)Social support(Japanese version of the Generic Job Stress Questionnaire, GJSQ)
100 [122]	Morimoto and Shimada (2014)	Cross-sectional	Private companies	737 employees from an information technology company	Psychological stress response (18 items)	Continuous score	Coping strategies Appraisal of coping acceptability (Coping Scale for Task Stressors and Job Evaluation Stressors, CSTJ; Coping Scale for Interpersonal Stressors, CSI) Motivation for using the chosen coping strategy (Reason for Selection of Coping Scale, RSC)
101 [123]	Maruya et al. (2014)	Cross-sectional	Nursing or welfare facilities	33 child-care supporters working at Children and Family Support Centers in four cities in Tokyo	Quantitative job overload (3 items) Qualitative job overload (3 items) Physical demands (1 item) Interpersonal conflict (3 items) Poor physical environment (1 item) Job control (3 items) Skill utilization (1 item) Suitable jobs (1 item) Meaningfulness of work (1 item) Vigor (3 items) Anger-irritability (3 items) Fatigue (3 items) Anxiety (3 items) Depression (6 items) Physical stress response (11 items)	Continuous score	Quality of life (26-item World Health Organization Quality of Life Questionnaire, WHO QOL26)
102 [124]	Ikeshita et al. (2014)	Cross-sectional	Hospitals	98 nurses working in operating rooms	Job control (3 items) Skill utilization (1 item) Suitable jobs (1 item) Meaningfulness of work (1 item) Vigor (3 items) Anger-irritability (3 items) Fatigue (3 items) Anxiety (3 items) Depression (6 items) Physical stress response (11 items) Job and life satisfaction (2 items)	The participants were dichotomized into high and low groups. For the psychological stress response, ≥14 and ≥13 indicated a high score in men and women, respectively. For the physical stress response, ≥5 and ≥6 indicated a high score in men and women, respectively. Standardized scores were developed on a five-point scale based on a representative sample of Japanese workers.	General self-efficacy (General Self-Efficacy Scale, GSES)
103 [125]	Yada et al. (2014)	Cross-sectional	Hospitals	60 psychiatric nurses	Vigor (3 items) Anger-irritability (3 items) Fatigue (3 items) Anxiety (3 items) Depression (6 items) Physical stress response (11 items)	Continuous score	Psychiatric nurse job stressor (Psychiatric Nurse Job Stressor Scale, PNJSS)
104 [126]	Sato (2014)	Cross-sectional	Hospitals	160 ward nurses from two hospitals in a prefecture	Poor physical environment (1 item) Depression (6 items) Physical stress response (11 items)	Continuous score	Work environment perception (Items based on the Home and Community Environment, HACE)
105 [127]	Kikuchi et al. (2014)	Cross-sectional	Hospitals	330 female nurses from a general hospital	Quantitative job overload (3 items) Job control (3 items) Supervisor and coworker support (6 items)	Continuous score	Depressive symptoms (Five-item screening from the Self-Rating Depression Scale and the Hospital Anxiety and Depression Scale)
106 [128]	Morimoto et al. (2014)	Cross-sectional	Private companies	738 employees in an information technology company	Psychological stress response (18 items)	Continuous score	Coping methods Appraisal of coping acceptability (Coping Scale for Task stressors and Job evaluation stressors, CSTJ; Coping Scale for Interpersonal stressors, CSI) Appraisal of a stressor’s controllability (Cognitive Appraisal Rating Scale, CARS)
107 [129]	Sugawara et al. (2013)	Cross-sectional	Private companies	5878 middle-aged workers randomly selected companies in the Aomori prefecture	Quantitative job overload (3 items) Qualitative job overload (3 items) Physical demands (1 item) Interpersonal conflict (3 items) Poor physical environment (1 item) Job control (3 items) Skill utilization (1 item) Suitable jobs (1 item) Job satisfaction (1 item)	Job demands were calculated by quantitative, qualitative, and physical demands. Job compatibility was calculated by skill utilization and suitable jobs. Each variable was dichotomized based on the number of agreements for the items	Suicidal ideation Depressive symptoms (Center for Epidemiologic Studies for Depression scale, CES-D)
108 [130]	Okuno et al. (2013)	Cross-sectional	Hospitals	284 nurses working from a general hospital	Quantitative job overload (3 items) Qualitative job overload (3 items) Physical demands (1 item) Job control (3 items) Interpersonal conflict (3 items) Poor physical environment (1 item) Skill utilization (1 item) Suitable jobs (1 item) Meaningfulness of work (1 item) Supervisor support (3 items) Coworker support (3 items) Support from family and friends (3 items) Job satisfaction (1 item) Satisfaction with family life (1 item)	Standardized scores were developed on a 5-point scale based on a representative sample of Japanese workers. For social support and satisfaction, continuous scores were used.	Post-traumatic growth (Japanese version of Posttraumatic Growth Inventory, PTGI-J) Burnout (Copenhagen Burnout Inventory, CBI)
109 [131]	Yoshida et al. (2013)	Cross-sectional	Hospitals	517 nurses from a university hospital	Psychological stress response (18 items) Physical stress response (11 items)	Standardized scores were developed on a five-point scale based on a representative sample of Japanese workers.	Sense of coherence(Japanese Sense of Coherence scale: SOC-13)Coping(Brief Scales for Coping Profile; BSCP)
110 [132]	Shigehisa (2013)	Cross-sectional	Hospitals	357 nurses	Quantitative job overload (3 items) Qualitative job overload (3 items) Physical demands (1 item) Interpersonal conflict (3 items) Poor physical environment (1 item) Job control (3 items) Skill utilization (1 item) Suitable jobs (1 item) Meaningfulness of work (1 item)	Continuous score	Caring behaviors (41-item caring behavior scale)
111 [133]	Koizumi et al. (2013)	Cross-sectional	Hospitals	225 female nurses from a general hospital	Vigor (3 items) Anger-irritability (3 items) Fatigue (3 items) Anxiety (3 items) Depression (6 items) Physical stress response (11 items)	Continuous score	Resilience (Sukemune-Hiew Resilience Test, SHR)
112 [134]	Hosoda et al. (2012)	Cross-sectional	Fire defense stations/headquarters	246 male firefighters from a local fire defense headquarters	Quantitative job overload (3 items) Qualitative job overload (3 items) Physical demands (1 item) Interpersonal conflict (3 items) Poor physical environment (1 item) Job control (3 items) Skill utilization (1 item) Suitable jobs (1 item) Meaningfulness of work (1 item) Vigor (3 items) Anger-irritability (3 items) Fatigue (3 items) Anxiety (3 items) Depression (6 items) Physical stress response (11 items) Supervisor support (3 items) Coworker support (3 items) Support from family and friends (3 items)	Continuous score	Alcohol dependence (Alcohol Use Disorders Identification Test, AUDIT)
113 [135]	Sunami and Yaeda (2012)	Cross-sectional	Hospitals	472 novice nurses from 15 hospitals with 300 or more beds	Psychological stress response (18 items)	Continuous score	Social support (Mentoring scale from Ono, 1998) Self-esteem (Rosenberg’s self-esteem scale)
114 [136]	Taniguchi et al. (2012)	Cross-sectional	Nursing or welfare facilities	897 care workers working in 35 nursing facilities	Vigor (3 items) Anger-irritability (3 items) Fatigue (3 items) Anxiety (3 items) Depression (6 items) Physical stress response (11 items) Psychological stress response (18 items)	Continuous score	Workplace bullying (Negative Acts Questionnaire, NAQ)
115 [137]	Amagasa and Nakayama (2012)	Cross-sectional	Private companies	1160 sales workers	Quantitative job overload (3 items) Qualitative job overload (3 items) Job control (3 items)	Continuous score	Depression (Center for Epidemiologic Studies for Depression scale, CES-D)
116 [138]	Hayashi et al. (2011)	Cross-sectional	Private companies	1804 full-time regular employees from an electronics company	Quantitative job overload (3 items) Job control (3 items) Supervisor and coworker support (6 items)	Continuous score	Organizational justice(Organizational Justice Questionnaire, OJQ)Organizational citizenship behavior(3-item scale based on Williams and Anderson, 1991)Job satisfaction(6-item scale from Tanaka, 1995)
117 [139]	Ugaki et al. (2010)	Cross-sectional	Nursing or welfare facilities	150 nurses who participated in the psychoeducation program in a prefecture	Interpersonal conflict (3 items) Poor physical environment (1 item) Job control (3 items) Suitable jobs (1 item) Vigor (3 items) Anger-irritability (3 items) Fatigue (3 items) Anxiety (3 items) Depression (6 items)	Continuous score	Coping traits (Brief Stress for Coping Scale, BSCP)
118 [140]	Katayama (2010)	Cross-sectional	Hospitals	123 nurses from five hospitals with 300 or more beds	Vigor (3 items) Anger-irritability (3 items) Fatigue (3 items) Anxiety (3 items) Depression (6 items) Physical stress response (11 items)	Continuous score	Emotional labor(Emotional Labor Inventory for Nurses, ELIN)
119 [141]	Shimazu et al. (2010)	Cross-sectional	Private companies	757 workers from a construction machinery company	Psychological stress response except for vigor (15 items) Physical stress response (11 items)	Continuous score	Workaholism (Dutch Workaholism Scale, DUWAS) Active coping (Brief Stress for Coping Scale, BSCP) Job performance (World Health Organization Health and Work Performance Questionnaire, WHO-HPQ)
120 [142]	Shimazu and Schaufeli (2009)	Cross-sectional	Private companies	776 employees from a construction machinery company	Psychological stress response (18 items) Physical stress response (11 items) Job satisfaction (1 item) Satisfaction with family life (1 item)	Continuous score	Work engagement (Utrecht Work Engagement Scale, UWES) Workaholism (Dutch Workaholism Scale, DUWAS) Job performance (World Health Organization Health and Work Performance Questionnaire, WHO-HPQ)
121 [143]	Otsuka et al. (2009)	Cross-sectional	Private companies	808 middle-aged workers from a company in Kanagawa Prefecture	Quantitative job overload (3 items) Qualitative job overload (3 items) Job control (3 items)	The total scores for the two scales were dichotomized at the median, and high job strain was defined as the combination of high job demands and low job control.	Arterial wave reflection (Automated applanation tonometric method)
122 [144]	Katsuyama et al. (2009)	Cross-sectional	Private companies	243 employees who worked at a manufacturing company and a local hospital	Depression (6 items)	Continuous score	Polymorphisms of the serotonin transporter (5HTT) Aldehyde dehydrogenase 2 (ALDH2) D2 dopamine receptor (DRD2) Cytochrome P450 2A6 (CYP2A6)
123 [145]	Sato et al. (2009)	Cross-sectional	Private companies	24,685 employees from a computer, software, and network company	Stress responses (29 items)	Continuous score based on a five-point scale	Overtime work (Self-reported)
124 [146]	Tanbo (2008)	Cross-sectional	Healthcare centers	62 workers who took a health examination at a health institute	Vigor (3 items) Anger-irritability (3 items) Fatigue (3 items) Anxiety (3 items) Depression (6 items) Physical stress response (11 items)	Continuous score	Health Practice Index (Morimoto’s Health Practical Index, HPI)
125 [147]	Mitani et al. (2008)	Cross-sectional	Fire defense stations/headquarters	128 firefighters from a fire department	Job stressors (17 items) Social support (9 items)	Continuous score	Symptoms of post-traumatic stress disorder (Japanese version of Impact Event Scale, IES-R-J).
126 [148]	Katsuyama et al. (2008)	Cross-sectional	Private companies	243 employees at a manufacturing company and a local hospital	Depression (6 items)	Continuous score	Serotonin transporter gene polymorphisms (5HTT, Leukocytes in blood sample)
127 [149]	Ikeda et al. (2008)	Cross-sectional	Hospitals	76 doctors and 285 female nurses working at a general hospital	Quantitative job overload (3 items) Job control (3 items) Supervisor support (3 items) Coworker support (3 items)	Continuous score	Mental health (30-item General Health Questionnaire, GHQ-30)
128 [150]	Katsuyama (2008)	Cross-sectional	Private companies	133 manufacturing workers and 113 hospital workers	Quantitative job overload (3 items) Job control (3 items) Supervisor support (3 items) Coworker support (3 items)	Health risk scores were calculated by the four subscales	Polymorphisms of the serotonin transporter (5HTT) Aldehyde dehydrogenase 2 (ALDH2) D2 dopamine receptor (DRD2)
129 [151]	Suwazono et al. (2008)	Cross-sectional	Private companies	3481 daytime employees from a steel company	Quantitative job overload (3 items) Qualitative job overload (3 items) Physical demands (1 item) Job control (3 items) Interpersonal conflict (3 items) Suitable jobs (1 item)	Job demands were defined as high when the level of job demands was scored six points or more. Job control, interpersonal conflicts, and suitable jobs were defined as unfavorable when the level of each subscale was two points or more.	Fatigue symptoms (Cumulative Fatigue Symptom Index, CFSI)
130 [152]	Umehara et al. (2007)	Cross-sectional	Hospitals	590 respondents who worked more than 35 h per week as a pediatrician	Quantitative job overload (3 items) Job control (3 items) Supervisor support (3 items) Coworker support (3 items)	Continuous score	Working hours (Self-reported) Work schedule (Self-reported, number of night duties in the past month, number of weekend duties in the past month, days with on-call duties in the past month, workdays with no overtime in the past month, days off with no work in the past month, days off with some work in the past month)
131 [153]	Mineyama et al. (2007)	Cross-sectional	Private companies	203 workers at a brewing company in the Kansai (west) region	Vigor (3 items) Anger-irritability (3 items) Fatigue (3 items) Anxiety (3 items) Depression (6 items) Psychological stress response (18 items)	Continuous score	Active listening attitude of supervisors (Active Listening Attitude Scale, ALAS)
132 [154]	Tanihara and Taguchi (2007)	Cross-sectional	Private companies	49 workers from a small-sized worksite	Quantitative job overload (3 items) Qualitative job overload (3 items) Physical demands (1 item) Interpersonal conflict (3 items) Poor physical environment (1 item) Job control (3 items) Skill utilization (1 item) Suitable jobs (1 item) Meaningfulness of work (1 item) Vigor (3 items) Anger-irritability (3 items) Fatigue (3 items) Anxiety (3 items) Depression (6 items) Physical stress response (11 items)	Continuous score	Burnout (Maslach Burnout Inventory, MBI)
133 [155]	Washizuka and Ikeo (2007)	Cross-sectional	Hospitals	175 female nurses working in shift time at a hospital	Quantitative job overload (3 items) Interpersonal conflict (3 items) Job control (3 items) Suitable jobs (1 item) Psychological stress response (18 items) Physical stress response (11 items) Supervisor and coworker support (6 items)	Simple scoring method	Breslow health-related behaviors (Seven-item Breslow’s health behaviors)
134 [156]	Ikeda et al. (2007)	Cross-sectional	Hospitals	405 female nurses working at a general hospital	Quantitative job overload (3 items) Job control (3 items) Supervisor support (3 items) Coworker support (3 items)	Continuous score	Mental health (30-item General Health Questionnaire, GHQ-30)
135 [157]	Toh et al. (2006)	Cross-sectional	Hospitals	144 novice nurses at a university hospital	Psychological stress response (18 items)	Continuous score	Ego state (Egogram)
136 [158]	Mitani et al. (2006)	Cross-sectional	Fire defense stations/headquarters	231 participants belonging to two fire departments	Job stressors (17 items) Social support (9 items)	Continuous score	Burnout (Maslach Burnout Inventory, MBI) Symptoms of post-traumatic stress disorder (Japanese version of Impact Event Scale, IES-R-J).
137 [159]	Ushiki et al. (2006)	Cross-sectional	Hospitals	316 female nurses from a hospital	Quantitative job overload (3 items) Job control (3 items) Supervisor support (3 items) Coworker support (6 items)	Continuous score	Depression Anxiety (Goldberg’s anxiety-depression scale)
138 [160]	Mitani and Shirakawa (2005)	Cross-sectional	Fire defense stations/headquarters	37 firefighters working at a fire department in Kyoto city	Job stressors (17 items)	Participants were dichotomized into high and low groups based on the mean score.	Autonomic nervous activity (Electro-cardiogram) Serum cortisol Norepinephrine (Blood sampling)
139 [161]	Harada et al. (2005)	Cross-sectional	Private companies	4962 male workers from a steel company	Quantitative job overload (3 items) Qualitative job overload (3 items) Physical demands (1 item) Job control (3 items) Interpersonal conflict (3 items) Suitable jobs (1 item)	Job demands were defined as unfavorable when six or more questions on job demands were ticked by a participant. Job control, interpersonal conflict, and suitable jobs were defined as unfavorable when two or more items were ticked for each item.	Shift work (Self-reported)
140 [162]	Shimazu et al. (2005)	Cross-sectional	Private companies	726 male non-managers at a large electrical company	Quantitative job overload (3 items) Job control (3 items) Supervisor support (3 items) Coworker support (3 items)	Continuous score	Active coping (Job Stress Scale, JSS)
141 [163]	Katsuyama et al. (2005)	Cross-sectional	Private companies	133 workers from a manufacturing company	Quantitative job overload (3 items) Job control (3 items) Supervisor support (3 items) Coworker support (3 items)	Health risk scores were calculated by the four subscales	Polymorphisms of the serotonin transporter (5HTT) Aldehyde dehydrogenase 2 (ALDH2)
142 [164]	Shimazu et al. (2004)	Cross-sectional	Private companies	867 employees from a large electrical company	Quantitative job overload (3 items) Job control (3 items) Supervisor support (3 items) Coworker support (3 items)	Continuous score	Job satisfaction (10-item scale from McLean, 1979)
143 [165]	Miki et al. (2004)	Cross-sectional	Hospitals	695 full-time nurses from a university hospital	Quantitative job overload (3 items) Qualitative job overload (3 items) Physical demands (1 item) Interpersonal conflict (3 items) Poor physical environment (1 item) Job control (3 items) Skill utilization (1 item) Suitable jobs (1 item) Supervisor support (3 items) Coworker support (3 items) Job satisfaction (1 item)	Continuous score	Depression (Center for Epidemiologic Studies for Depression scale, CES-D)
144 [166]	Tsukamoto et al. (2004)	Cross-sectional	Private companies	808 male workers from an informational technology company	Quantitative job overload (3 items) Job control (3 items) Supervisor support (3 items) Coworker support (3 items)	Continuous score	Depression (Zung Self-rating Depression Scale, SDS)
145 [167]	Kotake et al. (2003)	Cross-sectional	Hospitals	113 novice nurses at a university hospital	Quantitative job overload (3 items) Qualitative job overload (3 items) Physical demands (1 item) Job control (3 items) Suitable jobs (1 item)	Continuous score	Mental health (12-item General Health Questionnaire, GHQ-12)

## References

[1] Kivimäki M., Nyberg S.T., Batty G.D., Fransson E.I., Heikkilä K., Alfredsson L., Bjorner J.B., Borritz M., Burr H., Casini A. (2012). Job strain as a risk factor for coronary heart disease: A collaborative meta-analysis of individual participant data. Lancet.

[2] Kivimaki M., Virtanen M., Elovainio M., Kouvonen A., Vaananen A., Vahtera J. (2006). Work stress in the etiology of coronary heart diseas—A meta-analysis. Scand. J. Work Environ. Health.

[3] Stansfeld S., Candy B. (2006). Psychosocial work environment and mental health—A meta-analytic review. Scand. J. Work Environ. Health.

[4] World Health Organization (2008). PRIMA-EF: Guidance on the European Framework for Psychosocial Risk Management: A Resource for Employers and Worker Representatives. https://apps.who.int/iris/handle/10665/43966.

[5] Cousins R., MacKay C.J., Clarke S.D., Kelly C., Kelly P.J., McCaig R.H. (2004). ‘Management Standards’ work-related stress in the UK: Practical development. Work Stress.

[6] National Standard of Canada (2013). Psychological Health and Safety in the Workplace: Prevention, Promotion, and Guidance to Staged Implementation. https://www.healthandsafetybc.ca/wp-content/uploads/2016/04/CAN_CSA-Z1003-13_BNQ_9700-803_2013_EN.pdf.

[7] International Organization for Standardization (2021). ISO45003:2021 Occupational Health and Safety Management: Psychological Health and Safety at Work: Guidelines for Managing Psychosocial Risks. https://www.iso.org/standard/64283.html.

[8] Kristensen T.S., Hannerz H., Hogh A., Borg V. (2005). The Copenhagen Psychosocial Questionnaire—A tool for the assessment and improvement of the psychosocial work environment. Scand. J. Work Environ. Health.

[9] Pejtersen J.H., Kristensen T.S., Borg V., Bjorner J.B. (2010). The second version of the Copenhagen Psychosocial Questionnaire. Scand. J. Public Health.

[10] Burr H., Berthelsen H., Moncada S., Nubling M., Dupret E., Demiral Y., Oudyk J., Kristensen T.S., Llorens C., Navarro A. (2019). The third version of the Copenhagen Psychosocial Questionnaire. Saf. Health Work.

[11] Hurrell J.J., McLaney M.A. (1988). Exposure to job stress: A new psychometric instrument. Scand. J. Work Environ. Health.

[12] Chang S.J., Koh S.B., Kang D., Kim S.A., Kang M.G., Lee C.G., Chung J.J., Cho J.J., Son M., Chae C.H. (2005). Developing an Occupational Stress Scale for Korean employees. Korean J. Occup. Environ. Med..

[13] Wu X., Li Y., Yao Y., Luo X., He X., Yin W. (2018). Development of construction workers job stress scale to study and the relationship between job stress and safety behavior: An empirical study in Beijing. Int. J. Environ. Res. Public Health.

[14] Naono-Nagatomo K., Abe H., Yada H., Higashizako K., Nakano M., Takeda R., Ishida Y. (2019). Development of the School Teachers Job Stressor Scale (STJSS). Neuropsychopharmacol. Rep..

[15] Mert S., Aydin Sayilan A., Baydemir C. (2021). Nurse Stress Scale (NSS): Reliability and validity of the Turkish version. Perspect. Psychiatr. Care.

[16] Song K.W., Kim H.K. (2019). Job stress and its related factors among Korean dentists: An online survey study. Int. Dent. J..

[17] Shimomitsu T., Haratani T., Nakamura K., Kawakami N., Hyashi T., Hiro H., Arai M., Miyazaki S., Furuki K., Ohya Y., Kato M. (2000). Final development of the Brief Job Stress Questionnaire mainly used for assessment of the individuals. The Ministry of Labor Sponsored Grant for the Prevention of Work-Related Illness.

[18] Inoue A., Kawakami N., Shimomitsu T., Tsutsumi A., Haratani T., Yoshikawa T., Shimazu A., Odagiri Y. (2014). Development of a short questionnaire to measure an extended set of job demands, job resources, and positive health outcomes: The New Brief Job Stress Questionnaire. Ind. Health.

[19] Kawakami N., Tsutsumi A. (2016). The Stress Check Program: A new national policy for monitoring and screening psychosocial stress in the workplace in Japan. J. Occup. Health.

[20] Pousa P.C.P., Lucca S.R. (2021). Psychosocial factors in nursing work and occupational risks: A systematic review. Rev. Bras. Enferm..

[21] Fernandes C., Pereira A. (2016). Exposure to psychosocial risk factors in the context of work: A systematic review. Rev. Saude Publica.

[22] Page M.J., McKenzie J.E., Bossuyt P.M., Boutron I., Hoffmann T.C., Mulrow C.D., Shamseer L., Tetzlaff J.M., Akl E.A., Brennan S.E. (2021). The PRISMA 2020 statement: An updated guideline for reporting systematic reviews. BMJ.

[23] Sugito M., Okada Y., Torimoto K., Enta K., Tanaka Y. (2021). Work environment-related stress factors are correlated with diabetes development in workers with impaired glucose tolerance: A 5-year follow-up study using the Brief Job Stress Questionnaire (BJSQ). J. UOEH.

[24] Takahashi K., Kamino T., Yasuda T., Suganuma A., Sakane N. (2020). Association between psychological distress and stress-related symptoms and increased risk of type 2 diabetes in male individuals: An observational study. J. Clin. Med. Res..

[25] Kachi Y., Inoue A., Eguchi H., Kawakami N., Shimazu A., Tsutsumi A. (2020). Occupational stress and the risk of turnover: A large prospective cohort study of employees in Japan. BMC Public Health.

[26] Shimazaki T., Uechi H., Takenaka K. (2020). Mental health promotion behaviors associated with a 6-Month follow-up on job-related mood among Japanese workers. Int. Perspect. Psychol. Res. Pract. Consul..

[27] Wang W., Sakata K., Komiya A., Li Y. (2020). What makes employees’ work so stressful? Effects of vertical leadership and horizontal management on employees’ stress. Front. Psychol..

[28] Inoue A., Tsutsumi A., Kachi Y., Eguchi H., Shimazu A., Kawakami N. (2020). Psychosocial work environment explains the association of job dissatisfaction with long-term sickness absence: A one-year prospect study of Japanese employees. J. Epidemiol..

[29] Hino A., Inoue A., Mafune K., Hiro H. (2019). The effect of changes in overtime work hours on depressive symptoms among Japanese white-collar workers: A 2-year follow-up study. J. Occup. Health.

[30] Morimoto H., Furuta N., Kono M., Kabeya M. (2019). Stress-buffering effect of coping strategies on interrole conflict among family caregivers of people with dementia. Clin. Gerontol..

[31] Ogawa K., Iwasaki S., Deguchi Y., Fukuda Y., Nitta T., Nogi Y., Mitake T., Inoue K. (2018). Higher occupational stress and stress responses in public servants requiring long-term sickness absence due to mental disorders. Osaka City Med. J..

[32] Tsutsumi A., Shimazu A., Eguchi H., Inoue A., Kawakami N. (2018). A Japanese Stress Check Program screening tool predicts employee long-term sickness absence: A prospective study. J. Occup. Health.

[33] Fukuda Y., Iwasaki S., Deguchi Y., Ogawa K., Nitta T., Inoue K. (2018). The effect of long-term sickness absence on coworkers in the same work unit. Ind. Health.

[34] Okita S., Daitoku S., Abe M., Arimura E., Setoyama H., Koriyama C., Ushikai M., Kawaguchi H., Horiuchi M. (2017). Potential predictors of susceptibility to occupational stress in Japanese novice nurses—A pilot study. Environ. Health Prev. Med..

[35] Hino A., Inoue A., Mafune K., Nakagawa T., Hayashi T., Hiro H. (2016). Changes in the psychosocial work characteristics and insulin resistance among Japanese male workers: A three-year follow-up study. J. Occup. Health.

[36] Sakuraya A., Imamura K., Inoue A., Tsutsumi A., Shimazu A., Takahashi M., Totsuzaki T., Kawakami N. (2017). Workplace social capital and the onset of major depressive episode among workers in Japan: A 3-year prospective cohort study. J. Epidemiol. Community Health.

[37] Taniguchi T., Takaki J., Hirokawa K., Fujii Y., Harano K. (2016). Associations of workplace bullying and harassment with stress reactions: A two-year follow-up study. Ind. Health.

[38] Watanabe K., Otsuka Y., Shimazu A., Kawakami N. (2016). The moderating effect of health-improving workplace environment on promoting physical activity in white-collar employees: A multi-site longitudinal study using multi-level structural equation modeling. J. Occup. Environ. Med..

[39] Endo M., Muto T., Haruyama Y., Yuhara M., Sairenchi T., Kato R. (2015). Risk factors of recurrent sickness absence due to depression: A two-year cohort study among Japanese employees. Int. Arch. Occup. Environ. Health.

[40] Shimazu A., Schaufeli W.B., Kamiyama K., Kawakami N. (2015). Workaholism vs. work engagement: The two different predictors of future well-being and performance. Int. J. Behav. Med..

[41] Matsudaira K., Konishi H., Miyoshi K., Isomura T., Inuzuka K. (2014). Potential risk factors of persistent low back pain developing from mild low back pain in urban Japanese workers. PLoS ONE.

[42] Wada K., Sairenchi T., Haruyama Y., Taneichi H., Ishikawa Y., Muto T. (2013). Relationship between the onset of depression and stress response measured by the Brief Job Stress Questionnaire among Japanese employees: A cohort study. PLoS ONE.

[43] Demerouti E., Shimazu A., Bakker A.B., Shimada K., Kawakami N. (2013). Work-self balance: A longitudinal study on the effects of job demands and resources on personal functioning in Japanese working parents. Work Stress.

[44] Okuno Y., Banba I., Aono A., Azuma K., Okumura J. (2013). A longitudinal study on the effect of stress experiences on self-growth one year later among human service professionals. Med. J. Kinki. Univ..

[45] Urakawa K., Yokoyama K., Itoh H. (2012). Sense of coherence is associated with reduced psychological responses to job stressors among Japanese factory workers. BMC Res. Notes.

[46] Takahashi M., Iwasaki K., Sasaki T., Kubo T., Mori I., Otsuka Y. (2012). Sleep, fatigue, recovery, and depression after change in work time control: A one-year follow-up study. J. Occup. Environ. Med..

[47] Sugimura H., Thériault G. (2010). Impact of supervisor support on work ability in an IT company. Occup. Med..

[48] Shimazu A., de Jonge J. (2009). Reciprocal relations between effort-reward imbalance at work and adverse health: A three-wave panel survey. Soc. Sci. Med..

[49] Shimazu A., de Jonge J., Irimajiri H. (2008). Lagged effects of active coping within the demand-control model: A three-wave panel study among Japanese employees. Int. J. Behav. Med..

[50] Shimazu A., Schaufeli W.B. (2007). Does distraction facilitate problem-focused coping with job stress? A 1 year longitudinal study. J. Behav. Med..

[51] Tsuboi H., Tatsumi A., Yamamoto K., Kobayashi F., Shimoi K., Kinae N. (2006). Possible connections among job stress, depressive symptoms, lipid modulation and antioxidants. J. Affect. Disord..

[52] Hirokawa K., Ohira T., Nagao M., Nagayoshi M., Kajiura M., Imano H., Kitamura A., Kiyama M., Okada T., Iso H. (2022). Associations between occupational status, support at work, and salivary cortisol levels. Int. J. Behav. Med..

[53] Takaesu Y., Shimura A., Komada Y., Futenma K., Ishii M., Sugiura K., Watanabe K., Inoue Y. (2021). Association of sleep duration on workdays or free days and social jetlag with job stress. Psychiatry Clin. NeuroSci..

[54] Hidaka Y., Imamura K., Watanabe K., Tsutsumi A., Shimazu A., Inoue A., Hiro H., Odagiri Y., Asai Y., Yoshikawa T. (2021). Associations between work-related stressors and QALY in a general working population in Japan: A cross-sectional study. Int. Arch. Occup. Environ. Health.

[55] Toyoshima K., Inoue T., Shimura A., Masuya J., Fujimura Y., Higashi S., Kusumi I. (2021). The relationship among sleep reactivity, job-related stress, and subjective cognitive dysfunction: A cross-sectional study using path analysis. Ind. Health.

[56] Adachi H., Yamamoto R., Fujino R., Kanayama D., Sakagami Y., Akamine S., Marutani N., Yanagida K., Mamiya Y., Koyama M. (2021). Association of weekday-to-weekend sleep differences and stress response among a Japanese working population: A cross-sectional study. Sleep Med..

[57] Hayashida T., Shimura A., Higashiyama M., Fujimura Y., Ono K., Inoue T. (2021). Psychosomatic stress responses and sleep disturbance mediate the effects of irregular mealtimes on presenteeism. Neuropsychiatr Dis. Treat..

[58] Ôga Y., Chiba A. (2021). Relationship between nurses’ expression of humor toward their colleagues, and their physical and mental reactions and social support. Sangyo Eiseigaku Zasshi.

[59] Adachi K., Inaba R. (2021). Impacts of shift work on job stress and lifestyle. Jpn. J. Occup. Med. Traumatol..

[60] Ooka M., Odagiri Y., Kikuchi H., Takamiya T., Fukushima N., Hayashi T., Nakanishi Y., Shimomitsu T., Inoue S. (2019). Prevalence of and correlates with high stress among workers by job category according to Stress Check Program. J. Tokyo Med. Coll..

[61] Terada T., Nagamine M. (2020). The different influence of resilience and hardiness on the stress process in Japan Ground Self-Defense Force Service members. Jpn. J. Stress Sci..

[62] Sameshima H., Shimura A., Ono K., Masuya J., Ichiki M., Nakajima S., Odagiri Y., Inoue S., Inoue T. (2020). Combined effects of parenting in childhood and resilience on work stress in nonclinical adult workers from the community. Front. Psychiatry.

[63] Shimura A., Sugiura K., Inoue M., Misaki S., Tanimoto Y., Oshima A., Tanaka T., Yokoi K., Inoue T. (2020). Which sleep hygiene factors are important? Comprehensive assessment of lifestyle habits and job environment on sleep among office workers. Sleep Health.

[64] Furuichi W., Shimura A., Miyama H., Seki T., Ono K., Masuya J., Inoue T. (2020). Effects of job stressors, stress response, and sleep disturbance on presenteeism in office workers. Neuropsychiatr. Dis. Treat..

[65] Miyama H., Shimura A., Furuichi W., Seki T., Ono K., Masuya J., Odagiri Y., Inoue S., Inoue T. (2020). Association of chronotypes and sleep disturbance with perceived job stressors and stress response: A covariance structure analysis. Neuropsychiatr. Dis. Treat..

[66] Seki T., Shimura A., Miyama H., Furuichi W., Ono K., Masuya J., Odagiri Y., Inoue S., Inoue T. (2020). Influence of parenting quality and neuroticism on perceived job stressors and psychological and physical stress response in adult workers from the community. Neuropsychiatr. Dis. Treat..

[67] Kikuchi H., Odagiri Y., Ohya Y., Nakanishi Y., Shimomitsu T., Theorell T., Inoue S. (2020). Association of overtime work hours with various stress responses in 59,021 Japanese workers: Retrospective cross-sectional study. PLoS ONE.

[68] Taya H., Shimura A., Ishibashi Y., Misaki S., Inoue T. (2020). Presenteeism is Associated with Job Stress. Clin. Psychiatry.

[69] Hayasaki K., Shimabukuro E., Iguchi M., Mitsugasira S., Matsumoto M. (2020). Association between job stress and ethical behaviors among nurses. Kango Kanri.

[70] Saito Y., Ogawa M., Tanaka A. (2020). Association between generalized self-efficacy and stress among mid-career nursing teacher working at a training school for nurses in District A. Kango Kanri.

[71] Okamoto K., Tokizane R., Taniguchi T. (2020). Inappropriate care by livelihood support workers in support facilities for persons with disabilities and related factors. J. Health Welf. Stat..

[72] Nagata M., Nagata T., Inoue A., Mori K., Matsuda S. (2019). Effect modification by attention deficit hyperactivity disorder (ADHD) symptoms on the association of psychosocial work environments with psychological distress and work engagement. Front. Psychiatry.

[73] Okawa N., Kuratsune D., Koizumi J., Mizuno K., Kataoka Y., Kuratsune H. (2019). Application of autonomic nervous function evaluation to job stress screening. Heliyon.

[74] Maeda E., Nomura K., Hiraike O., Sugimori H., Kinoshita A., Osuga Y. (2019). Domestic work stress and self-rated psychological health among women: A cross-sectional study in Japan. Environ. Health Prev. Med..

[75] Sakamoto Y., Oka T., Amari T., Simo S. (2019). Factors affecting psychological stress in healthcare workers with and without chronic pain: A cross-sectional study using multiple regression analysis. Medicina.

[76] Matsumoto Y., Yoshioka S.I. (2019). Factors influencing psychiatric nurses’ job satisfaction levels: Focusing on their frequency of experiencing negative emotions toward patients and support at their workplaces. Yonago Acta Med..

[77] Kurebayashi Y. (2019). Comparison of factors predicting nursing skills between general and psychiatric nurses. Perspect. Psychiatr. Care.

[78] Watanabe M., Yamauchi K. (2019). Subtypes of overtime work and nurses’ fatigue, mental status, and work engagement: A latent class analysis of Japanese hospital nurses. J. Adv. Nurs..

[79] Fukunaga H., Yoshida H., Shimada R. (2019). Association between occupational stress and occupational identities: The analysis based on a questionnaire survey of nursing faculty members. J. Health Sci. Mind Body.

[80] Yoneyama M., Yoshida H., Kagitani F. (2019). Relationship between occupational stress and recognition: From a questionnaire survey of ward nurses. J. Health Sci. Mind Body.

[81] Inaba R. (2018). Study on the relationships between subjetive well-being and working condition, lifestyle or work-related stress among feale nurses. J. Jpn. Health Med. Assoc..

[82] Sato K. (2018). Factors affecting peridontal diseases and occupational stress in workers examination of health guidance based on saliva tests for peridontal diseases and assessment of salivary amylase levels. J. Jpn. Mibyo Syst. Assoc..

[83] Horie T., Yamano H., Hayami T., Tsuboi H., Nakajima Y. (2018). Association between job stress and burnout among teachers who train care workers. Kaigo Fukushi Kyouiku.

[84] Nakamura S., Mizukami K. (2018). Research on jon stress and individual factors of coventional type / unit type care workers: Comparative study of items related to job stress and stress response. J. Gerontol. Nurs. Caring Res..

[85] Enoki M., Maeda E., Iwata T., Murata K. (2018). Association between job stress and sleep time among call center employees. Akita J. Public Health.

[86] Okada N., Nakata A., Nakano M., Sakai K., Takai K., Kodama H., Kobayashi T. (2018). Stressors and the sense of coherence related to the mental health of nurses assuming the roles of wives and/or mothers—Investigation into the effects of leaving jobs because of marriage, childbirth, and childrearing. J. UOEH.

[87] Adachi K., Inaba R. (2018). Verification of the correlation of criteria for identifying highly stressed individuals based on the sum of psychological and physical stress reaction scores of the Brief Job Stress Questionnaire with the Center for Epidemiologic Studies Depression scale score. Jpn. J. Occup. Med. Traumatol..

[88] Sakamoto Y., Amari T., Shimo S. (2018). The relationship between pain psychological factors and job stress in rehabilitation workers with or without chronic pain. Work.

[89] Yada H., Abe H., Omori H., Ishida Y., Katoh T. (2017). Job-related stress in psychiatric assistant nurses. Nurs. Open.

[90] Enoki M., Maeda E., Iwata T., Murata K. (2017). The Association between work-related stress and autonomic imbalance among call center employees in Japan. Tohoku J. Exp. Med..

[91] Tsutsumi A., Inoue A., Eguchi H. (2017). How accurately does the Brief Job Stress Questionnaire identify workers with or without potential psychological distress?. J. Occup. Health.

[92] Saijo Y., Yoshioka E., Nakagi Y., Kawanishi Y., Hanley S.J.B., Yoshida T. (2017). Social support and its interrelationships with demand-control model factors on presenteeism and absenteeism in Japanese civil servants. Int. Arch. Occup. Environ. Health.

[93] Sakuraya A., Shimazu A., Eguchi H., Kamiyama K., Hara Y., Namba K., Kawakami N. (2017). Job crafting, work engagement, and psychological distress among Japanese employees: A cross-sectional study. Biopsychosoc. Med..

[94] Toyama H., Mauno S. (2017). Associations of trait emotional intelligence with social support, work engagement, and creativity in Japanese eldercare nurses. Jpn. Psychol. Res..

[95] Izawa S., Matsudaira K., Miki K., Arisaka M., Tsuchiya M. (2017). Psychosocial correlates of cortisol levels in fingernails among middle-aged workers. Stress.

[96] Watanabe M., Shimazu A., Bakker A.B., Demerouti E., Shimada K., Kawakami N. (2017). The impact of job and family demands on partner’s fatigue: A study of Japanese dual-earner parents. PLoS ONE.

[97] Yoshimoto T., Oka H., Katsuhira J., Fujii T., Masuda K., Tanaka S., Matsudaira K. (2017). Prognostic psychosocial factors for disabling low back pain in Japanese hospital workers. PLoS ONE.

[98] Sakagami Y. (2016). Qualitative job stress and ego aptitude in male scientific researchers. Work.

[99] Yamada K., Matsudaira K., Imano H., Kitamura A., Iso H. (2016). Influence of work-related psychosocial factors on the prevalence of chronic pain and quality of life in patients with chronic pain. BMJ Open.

[100] Otsuka Y., Nakata A., Sakurai K., Kawahito J. (2016). Association of suicidal ideation with job demands and job resources: A large cross-sectional study of Japanese workers. Int. J. Behav. Med..

[101] Watanabe K., Otsuka Y., Inoue A., Sakurai K., Ui A., Nakata A. (2016). Interrelationships between job resources, vigor, exercise habit, and serum lipids in Japanese employees: A multiple group path analysis using medical checkup data. Int. J. Behav. Med..

[102] Adachi K., Inaba R. (2016). Correlation between the Kessler Psychological Distress Scale and the criteria for identifying highly stressed individuals used in the Brief Job Stress Questionnaire. Bul Shubun Univ..

[103] Kawahito J. (2016). Self-complexity and depression in male middle-aged workers. Bul Hijiyama Univ..

[104] Maruya M., Tanaka Y. (2016). The condition of job stresses among care workers in facility for persons with intellectual disabilities and the relevance of the stresses to their QOL (quality of life). Syakai Fukushi Kagaku Kenkyu.

[105] Fujita S., Kawakami N., Ando E., Inoue A., Tsuno K., Kurioka S., Kawachi I. (2016). The association of workplace social capital with work engagement of employees in health care settings: A multilevel cross-sectional analysis. J. Occup. Environ. Med..

[106] Saijo Y., Chiba S., Yoshioka E., Nakagi Y., Ito T., Kitaoka-Higashiguchi K., Yoshida T. (2015). Synergistic interaction between job control and social support at work on depression, burnout, and insomnia among Japanese civil servants. Int. Arch. Occup. Environ. Health.

[107] Morimoto H., Shimada H., Tanaka H. (2015). Coping orientation and psychological distress in healthcare professionals: The utility of appraising coping acceptability. Jpn. Psychol. Res..

[108] Kagata S., Inoue A., Kubota K., Shimazu A. (2015). Association of emotional labor with work engagement and stress responses among hospital ward nurses. Jpn. J. Behav. Med..

[109] Lee G. (2015). Job stress and mental health among high-skilled foreign workers (HFWs) in Japan: Comparative analysis between HFWs and Japanese workers. Stress Sci. Res..

[110] Kato C., Shimada J., Hayashi K. (2015). Association between sleepiness and job stress among nurses. Jpn. J. Public Health.

[111] Inaba R., Inoue M. (2015). Relationship between burnout and work-related stress among female nurses report 2. Jpn. J. Occup. Med. Traumatol..

[112] Nakamura S., Mizukami K. (2015). Job stress and sense of coherence in care workers. J. Gerontol. Nurs. Caring Res..

[113] Igarashi H., Iijima S. (2015). Relation between QOL and occupational stress in female employee -Examining the differences between regular and non-regular employee-. Yamanashi Nurs. J..

[114] Ohta M., Higuchi Y., Yamato H., Kumashiro M., Sugimura H. (2015). Sense of coherence modifies the effect of overtime work on mental health. J. Occup. Health.

[115] Saijo Y., Chiba S., Yoshioka E., Kawanishi Y., Nakagi Y., Itoh T., Sugioka Y., Kitaoka-Higashiguchi K., Yoshida T. (2014). Effects of work burden, job strain and support on depressive symptoms and burnout among Japanese physicians. Int. J. Occup. Med. Environ. Health.

[116] Horita Y., Otsuka Y. (2014). Relationships between workers’ interpersonal helping behavior, social supports, job stressors, psychological stress responses, and vigor in manufacturing industry. Sangyo Eiseigaku Zasshi.

[117] Matsuzaki K., Uemura H., Yasui T. (2014). Associations of menopausal symptoms with job-related stress factors in nurses in Japan. Maturitas.

[118] Yoshida E., Yamada K., Morioka I. (2014). Sense of coherence (SOC), occupational stress reactions, and the relationship of SOC with occupational stress reactions among male nurses working in a hospital. Sangyo Eiseigaku Zasshi.

[119] Yada H., Abe H., Omori H., Matsuo H., Masaki O., Ishida Y., Katoh T. (2014). Differences in job stress experienced by female and male Japanese psychiatric nurses. Int. J. Ment. Health Nurs..

[120] Kikuchi Y., Nakaya M., Ikeda M., Okuzumi S., Takeda M., Nishi M. (2014). Sense of coherence and personality traits related to depressive state. Psychiatry J..

[121] Nakata A., Irie M., Takahashi M. (2014). Source-specific social support and circulating inflammatory markers among white-collar employees. Ann. Behav. Med..

[122] Morimoto H., Shimada H. (2015). The relationship between psychological distress and coping strategies: Their perceived acceptability within a socio-cultural context of employment, and the motivation behind their choices. Int. J. Stress Manag..

[123] Maruya M., Tanaka Y., Nakashima Y. (2014). The Condition of job stresses among child-care supporters at Children and Family Support Centers, and the relevance of the stresses to their quality of life (QOL). Syakai Fukushi Kagaku Kenkyu.

[124] Ikeshita D., Asa N., Shirakawa T., Kawasaki T., Mikami Z. (2014). Feature of operating room nurses general self efficacy and connection with stress. Med. J. Shikoku Med. Cent. Child. Adults.

[125] Yada H., Abe H., Kato T., Omori H., Ishida Y. (2014). The influence of job-related stressors on stress reactions in psychiatric recuperation ward nurses. Clin. Psychiatry.

[126] Sato Y. (2014). The relation of the environmental perception of a ward institution and stress in nurse’s. J. Med. Welf..

[127] Kikuchi Y., Nakaya M., Ikeda M., Okuzumi S., Takeda M., Nishi M. (2014). Relationship between job stress, temperament and depressive symptoms in female nurses. Int. J. Occup. Med. Environ. Health.

[128] Morimoto H., Shimada H., Ozaki K. (2014). Sociocultural beliefs, as well as goodness of fit, influence the effectiveness of coping in Japanese workers. Int. J. Behav. Med..

[129] Sugawara N., Yasui-Furukori N., Sasaki G., Tanaka O., Umeda T., Takahashi I., Danjo K., Matsuzaka M., Kaneko S., Nakaji S. (2013). Gender differences in factors associated with suicidal ideation and depressive symptoms among middle-aged workers in Japan. Ind. Health.

[130] Okuno Y., Banba I., Aono A., Azuma K., Okumura J. (2013). Sense of self-growth and burnout in hospital nurses. Jpn. J. Health Psychol..

[131] Yohida E., Yamada K., Shibataki H., Morioka I. (2013). Relationship between sense of coherence and stress reactions among nurses in a hospital. J. Jpn. Soc. Nurs. Res..

[132] Shigehisa K. (2013). Analysis of stress factors that influence the practice of caring behaviors important to oncology nursing. Jpn. J. Clin. Nurs. Mon..

[133] Koizumi H., Kawano A., Sankai C., Saeki Y. (2013). Demographic factors, resilience, and occupational stress symptoms among female nurses in Japan. Med. Biol..

[134] Hosoda T., Osaki Y., Okamoto H., Wada T., Otani S., Mu H.Y., Yokoyama Y., Okamoto M., Kurozawa Y. (2012). Evaluation of relationships among occupational stress, alcohol dependence and other factors in male personnel in a Japanese local fire fighting organization. Yonago Acta Med..

[135] Sunami N., Yaeda J. (2012). Association between types of support received by new nurses, self-esteem, and mental health. Kango Kanri.

[136] Taniguchi T., Takaki J., Harano K., Hirokawa K., Takahashi K., Fukuoka E. (2012). Associations between workplace bullying, harassment, and stress reactions of professional caregivers at welfare facilities for the elderly in Japan. Sangyo Eiseigaku Zasshi.

[137] Amagasa T., Nakayama T. (2012). Relationship between long working hours and depression in two working populations: A structural equation model approach. J. Occup. Environ. Med..

[138] Hayashi T., Odagiri Y., Ohya Y., Tanaka K., Shimomitsu T. (2011). Organizational justice, willingness to work, and psychological distress: Results from a private Japanese company. J. Occup. Environ. Med..

[139] Ugaki M., Miki A., Kawamoto S., Senoo A. (2010). Coping trait, stressors, and psychological stress reactions among nurses. Psychiatr. Ment. Health Nurs..

[140] Katayama H. (2010). Relationship between emotional labor and job-related stress among hospital nurses. Jpn. J. Hyg..

[141] Shimazu A., Schaufeli W.B., Taris T.W. (2010). How does workaholism affect worker health and performance? The mediating role of coping. Int. J. Behav. Med..

[142] Shimazu A., Schaufeli W.B. (2009). Is workaholism good or bad for employee well-being? The distinctiveness of workaholism and work engagement among Japanese employees. Ind. Health.

[143] Otsuka T., Kawada T., Ibuki C., Kusama Y. (2009). Relationship between job strain and radial arterial wave reflection in middle-aged male workers. Prev. Med..

[144] Katsuyama H., Tomita M., Okuyama T., Hidaka K., Watanabe Y., Tamechika Y., Fushimi S., Saijoh K. (2009). 5HTT polymorphisms are associated with job stress in Japanese workers. Leg. Med..

[145] Sato Y., Miyake H., Thériault G. (2009). Overtime work and stress response in a group of Japanese workers. Occup. Med..

[146] Tanbo K. (2008). Factor structure of health practice and the stress response in employees undertaking mandatory workplace check-ups. J. Tsuruma Health Sci. Soc..

[147] Mitani S. (2008). Comparative analysis of the Japanese version of the revised impact of event scale: A study of firefighters. Prehosp. Disaster Med..

[148] Katsuyama H., Tomita M., Hidaka K., Fushimi S., Okuyama T., Watanabe Y., Tamechika Y., Otsuki T., Saijoh K., Sunami S. (2008). Association between serotonin transporter gene polymorphisms and depressed mood caused by job stress in Japanese workers. Int. J. Mol. Med..

[149] Ikeda M., Nakaya M., Nishi M., Kikuchi Y., Kataoka M., Narita K. (2008). Mental health and job stress of medical staffs in a general hospital. Jpn. Bull. Soc. Psychiatry.

[150] Katsuyama H. (2008). Effects of stress in the workplace and stress-related polymorphisms on health. Occup. Health J..

[151] Suwazono Y., Dochi M., Kobayashi E., Oishi M., Okubo Y., Tanaka K., Sakata K. (2008). Benchmark duration of work hours for development of fatigue symptoms in Japanese workers with adjustment for job-related stress. Risk Anal..

[152] Umehara K., Ohya Y., Kawakami N., Tsutsumi A., Fujimura M. (2007). Association of work-related factors with psychosocial job stressors and psychosomatic symptoms among Japanese pediatricians. J. Occup. Health.

[153] Mineyama S., Tsutsumi A., Takao S., Nishiuchi K., Kawakami N. (2007). Supervisors’ attitudes and skills for active listening with regard to working conditions and psychological stress reactions among subordinate workers. J. Occup. Health.

[154] Tabihra H., Taguchi T. (2007). The relationship among “stressor”, “stress response” and “burnout syndrome” indices in small and medium sized enterprises. Kawasaki Med. Welf. J..

[155] Washizuka H., Ikeo K. (2006). Research on the association between daily health habits and job stress among nurses. Kango Kanri.

[156] Ikeda M., Nakaya M., Nishi M., Kataoka M., Horikawa N., Yamazaki T. (2007). Mental health of medical staff in a general hospital and Brief—JSQ scores. Jpn. Bull. Soc. Psychiatry.

[157] Toh S., Babazono A., Araki T. (2006). Association between the ego state and psychological stress responses of new nurses. Jpn. J. Health Care Manag. Market..

[158] Mitani S., Fujita M., Nakata K., Shirakawa T. (2006). Impact of post-traumatic stress disorder and job-related stress on burnout: A study of fire service workers. J. Emerg. Med..

[159] Ushiki A., Asai M., Nagata C., Nishimoto Y., Sato M., Goto K. (2005). Job stress among hospital nurses. -Association between nursing jobs, stress, and social support-. Kango Kanri.

[160] Mitani S., Shirakawa T. (2005). The relation between stress levels and heart rate variability among firefighters, paramedical and rescue service personnel. J. Disaster Med..

[161] Harada H., Suwazono Y., Sakata K., Okubo Y., Oishi M., Uetani M., Kobayashi E., Nogawa K. (2005). Three-shift system increases job-related stress in Japanese workers. J. Occup. Health.

[162] Shimazu A., Shimazu M., Odara T. (2005). Divergent effects of active coping on psychological distress in the context of the job demands-control-support model: The roles of job control and social support. Int. J. Behav. Med..

[163] Katsuyama H., Hidaka K., Tomita M., Watanabe Y., Okuyama T., Tamechika Y., Fushimi S., Sunami S. (2005). Interation between job stress and stress-related gene polymorphisms. DNA Polymorph..

[164] Shimazu A., Shimazu M., Odahara T. (2004). Job control and social support as coping resources in job satisfaction. Psychol. Rep..

[165] Miki A., Umezi C., Kanasaki Y. (2004). Job stress and mental health among Nurses -Study using the Brief Job Stress Questionnaire-. Psychiatr. Ment. Health Nurs..

[166] Tsukamoto K., Kikuchi H., Miyaska K. (2004). Relationship between job stressor, depressive symptom and workstyle for creative solution. Hitachi Med. J..

[167] Kotake Y., Fukuda N., Fukumoto H., Yoneta H., Kimura Y., Takahashi M., Takagawa S., Uchida S., Akatsuki A., Nagata S. (2003). A stress survey among novice nurses. Kango Kanri.

[168] Eguchi H., Tsuda Y., Tsukahara T., Washizuka S., Kawakami N., Nomiyama T. (2012). The effects of workplace occupational mental health and related activities on psychological distress among workers: A multilevel cross-sectional analysis. J. Occup. Environ. Med..

[169] Kawada T., Otsuka T. (2014). Change in job stress and job satisfaction over a two-year interval using the Brief Job Stress Questionnaire. Work.

[170] Iguchi A. (2016). Job demand and job resources related to the turnover intention of public health nurses: An analysis using a job demands-resources model. Jpn. J. Public Health.

[171] Huang Y., Yao D., Zhou H., Xi X., Wang Y., Yao W. (2021). Association of hospital pharmacy-related knowledge and skills with occupational stress of clinical pharmacists in tertiary hospitals of China. J. Am. Pharm. Assoc..

[172] Malcolm N., Boyd L., Giblin-Scanlon L., Vineyard J. (2020). Occupational stressors of dental hygienists in the United States. Work.

[173] Shinde V.V. (2019). Relationship of body mass index to job stress and eating behaviour in health care professionals-an observational study. Obes. Med..

[174] Karasek R.A. (1979). Job demands, job decision latitude, and mental strain: Implications for job redesign. Admin. Sci. Quart..

[175] Karasek R., Brisson C., Kawakami N., Houtman I., Bongers P., Amick B. (1998). The Job Content Questionnaire (JCQ): An instrument for internationally comparative assessments of psychosocial job characteristics. J. Occup. Health Psychol..

[176] Tokyo Medical University About the Brief Job Stress Questionnnaire. http://www.tmu-ph.ac/news/data/sotenkansan.pdf.

[177] Department of Digital Mental Health, Graduate School of Medicine, The University of Tokyo Scoring Methods of the Brief Job Stress Questionnaie. https://mental.m.u-tokyo.ac.jp/old/BJSQ/scoring.htm.

[178] Yokoyama K. (2021). A Study on Development of the Foreign Language Version of the Brief Job Stress Questionnaire. Research Report from 2018 to 2020 Granted by the Industrial Disease Clinical Research. https://www.mhlw.go.jp/content/000811554.pdf.

[179] Hidaka Y., Watanabe K., Imamura K., Tatha O., Kawakami N. (2022). Reliability and validity of the Chinese version of the New Brief Job Stress Questionnaire (New BJSQ) among workers in China. Ind. Health.

[180] Yan T., Ji F., Bi M., Wang H., Cui X., Liu B., Niu D., Li L., Lan T., Xie T. (2022). Occupational stress and associated risk factors among 13,867 industrial workers in China. Front. Public Health.

